# The Use of Advanced Mass Spectrometry to Dissect the Life-Cycle of Photosystem II

**DOI:** 10.3389/fpls.2016.00617

**Published:** 2016-05-10

**Authors:** Daniel A. Weisz, Michael L. Gross, Himadri B. Pakrasi

**Affiliations:** ^1^Department of Biology, Washington University in St. LouisSt. Louis, MO, USA; ^2^Department of Chemistry, Washington University in St. LouisSt. Louis, MO, USA

**Keywords:** Photosystem II, Photosystem II life-cycle, mass spectrometry, post-translational modification, chemical cross-linking, protein footprinting

## Abstract

Photosystem II (PSII) is a photosynthetic membrane-protein complex that undergoes an intricate, tightly regulated cycle of assembly, damage, and repair. The available crystal structures of cyanobacterial PSII are an essential foundation for understanding PSII function, but nonetheless provide a snapshot only of the active complex. To study aspects of the entire PSII life-cycle, mass spectrometry (MS) has emerged as a powerful tool that can be used in conjunction with biochemical techniques. In this article, we present the MS-based approaches that are used to study PSII composition, dynamics, and structure, and review the information about the PSII life-cycle that has been gained by these methods. This information includes the composition of PSII subcomplexes, discovery of accessory PSII proteins, identification of post-translational modifications and quantification of their changes under various conditions, determination of the binding site of proteins not observed in PSII crystal structures, conformational changes that underlie PSII functions, and identification of water and oxygen channels within PSII. We conclude with an outlook for the opportunity of future MS contributions to PSII research.

## Introduction

Since the late 1990s, mass spectrometry (MS) has become a central tool for the study of proteins and their role in biology. The advent of electrospray ionization (ESI) and matrix-assisted laser desorption ionization (MALDI) permits the ionization of peptides and proteins and their introduction into the gas phase, enabling their analysis by MS. The typical “bottom-up” workflow that emerged in the wake of these breakthroughs involves: (1) enzymatic digestion (often by trypsin) of a protein to produce peptides of small enough size (typically 1–3 kDa) to be ionized and fragmented efficiently in a mass spectrometer; (2) liquid chromatographic (LC) separation of the peptides; and (3) online (or offline) injection of the separated peptides into a mass spectrometer. The “top-down” approach is an attractive alternative that eliminates the protein digestion step, but the subsequent steps are generally more difficult for intact proteins than peptides, and this approach is currently best-suited for small, soluble proteins. After injection of the peptides, a typical tandem MS analysis consists of: (1) ionization of the peptide sample by ESI or MALDI and introduction into the gas phase; (2) measurement of the mass-to-charge (*m/z*) ratio of the intact peptide (also referred to as “MS^1^” analysis); and (3) fragmentation of the precursor ion and measurement of its “product-ion” spectrum (“MS/MS” or “MS^2^” analysis), which provides information about the peptide's amino acid sequence. When genomic information is available to predict the sequence of all proteins in the organism, computer analysis of the peptide masses and product-ion spectra can determine the highest-scoring match for each peptide from the protein database. This highest-scoring match is taken as the identity of the peptide, assuming data quality meets certain statistical criteria. A given protein is then determined to have been present in the sample if the quality and number of its peptide hits meet an additional set of statistical criteria. The ability to identify many proteins in a sample at once by MS has become the cornerstone of the field of proteomics.

Protein identification is only the most basic application of MS-based proteomics, and it has traditionally been described as the first “pillar” of the field. The second pillar is characterization of the many proteoforms that exist for each protein, arising, e.g., from splice variants and post-translational modifications (PTMs). These two pillars address questions about the *composition* of a protein sample. The third pillar is quantification—either absolute or relative—of proteins using isotopic labeling or label-free approaches. This pillar is typically used to address questions about the *dynamics* of a system—how composition of proteins or proteoforms changes over time, space, or under different environmental conditions or perturbations. A proposed fourth pillar focuses on the emerging area of structural proteomics that uses MS-based techniques to address questions about the three-dimensional *structure* of proteins and protein complexes in a cell.

**Figure 1:**
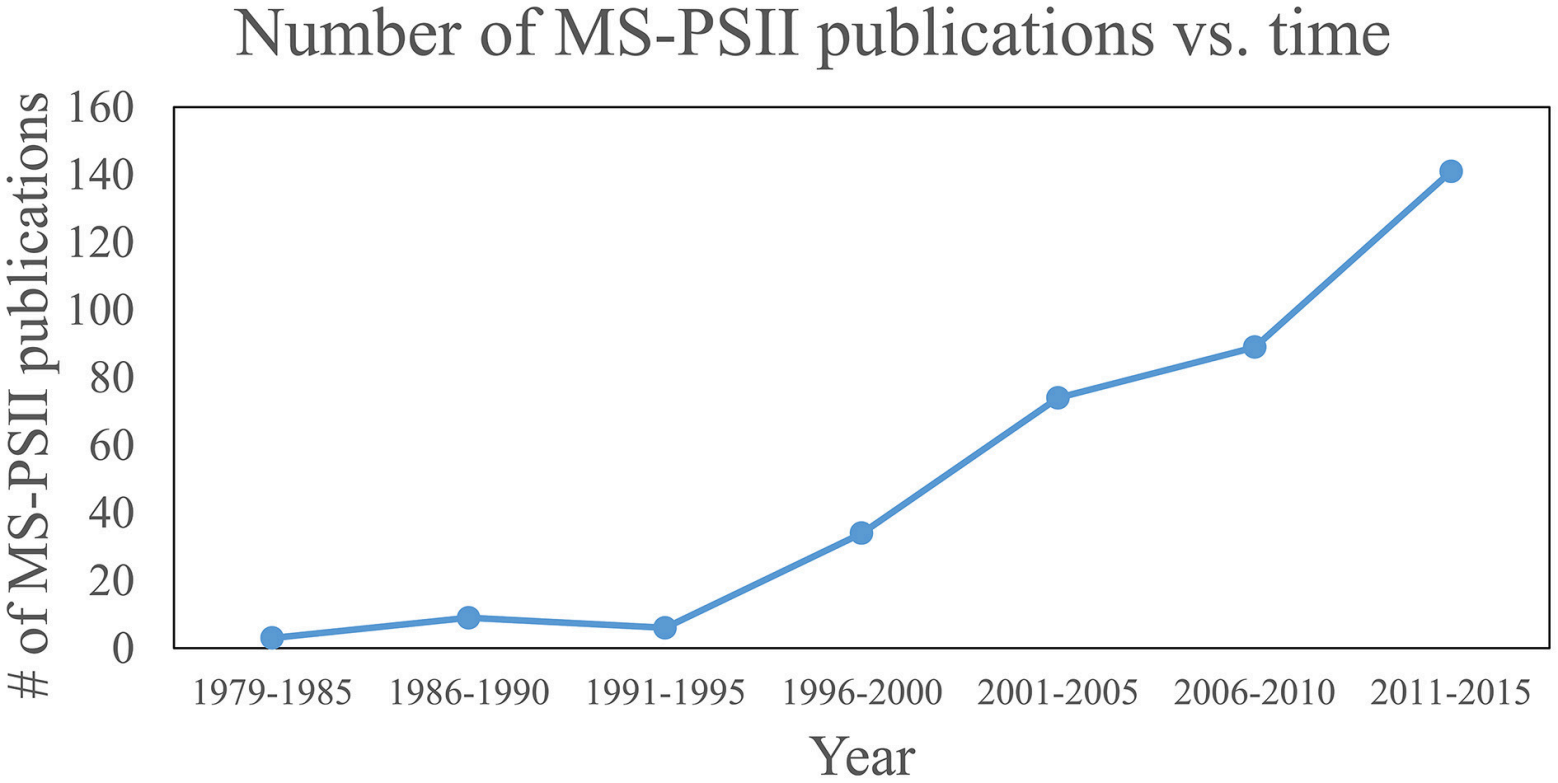
**Plot of publications that use MS for PSII research over time**. Publications that contain “Photosystem II” and “mass spectrometry” in their article title, abstract, or keywords were searched on the Scopus database. Each data point represents the total number of publications for that range of years.

These four pillars of proteomics have each become indispensable tools for gleaning information about photosynthesis (Battchikova et al., 2015; Bricker et al., 2015; Heinz et al., 2016) and, in particular for this review, the life-cycle of PSII. A search for publications containing both “Photosystem II” and “mass spectrometry” in the article title, abstract, and/or keywords was performed on the Scopus database. The results, displayed in Figure 1, show that prior to the advent of ESI and MALDI in the late 1980s, publications were nearly zero per year. Starting in the early 1990s and continuing through 2015, publications have risen steadily, with around 20–30 publications per year in the last several years. The rise can be attributed to method and instrument development, and to increasing accessibility of MS instrumentation to biology researchers. An overview of how MS-based tools are typically applied to PSII life-cycle research is given in Table 1. This review focuses on MS of proteins. However, it should be noted that another widely used application of MS in PSII research is the analysis of the isotopic composition of evolved oxygen by membrane-inlet mass spectrometry. This technique has yielded significant insight into the mechanistic aspects of water oxidation by PSII (reviewed in Shevela and Messinger, 2013).

**Table 1:** **Overview of the role of MS in PSII life-cycle research**.

**Kind of information**	**Information desired**	**MS-based technique**
Composition	PSII subunits present in a complex	Bottom-up MS (intact or top-down MS for LMM subunits)
	Accessory proteins that associate with PSII	Bottom-up MS
	PTMs	Bottom-up MS
Dynamics	Protein and PTM changes between samples	Label-free or isotopic-label-based relative quantification
	PSII subunit lifetime	Rate of unlabeled protein disappearance after isotopic label exposure
	Relative position of subcomplexes in PSII life-cycle	Relative isotopic label incorporation after pulse
Structure	Binding site of proteins not found in PSII crystal structures	Cross-linking, footprinting
	Conformational changes	Footprinting, quantify changes in modification extent
	Water and oxygen channel detection	Footprinting

In the sections that follow, we consider questions of PSII composition, dynamics, and structure separately. For each area, a brief overview of the relevant MS-based tools is given, followed by examples of several PSII life-cycle research areas that have benefitted from these techniques. In the final section, the outlook for future contributions of MS techniques to PSII life-cycle research is discussed.

## Composition of PSII complexes

### MS-based methods to study the composition of PSII complexes

#### PSII subunits with soluble domains

The bottom-up MS workflow is highly effective at identifying soluble proteins or proteins with soluble domains. It is, therefore, the main MS strategy that has been used to detect the core PSII proteins D1, D2, CP43, and CP47, which are transmembrane proteins but have multiple soluble domains, the extrinsic (soluble) PSII proteins, or unknown PSII-bound proteins. Bottom-up MS analysis can be preceded by either in-gel or in-solution digestion of the protein, each with advantages. Gel electrophoresis serves as a one- or two-dimensional fractionation step, simplifying the mixture to be analyzed by MS. Using this approach to remove interferences can improve instrument sensitivity toward proteins in the band of interest. Native PAGE, either alone or followed by denaturing SDS-PAGE (2D-BN-PAGE), is a common choice for resolving multiple protein complexes in a thylakoid membrane or purified PSII preparation; unknown bands can be excised and analyzed by MS to identify components of specific complexes (Granvogl et al., 2008; Pagliano et al., 2014; Gao et al., 2015). However, targeted band excision can miss potentially important proteins that migrated at positions not selected for in-gel digestion. In theory, native PAGE can remove unbound proteins from complexes, simplifying MS analysis; however, disruption of certain relevant protein-protein interactions in complexes cannot ever be fully excluded. Alternatively, in-solution digestion allows a more comprehensive analysis of the protein components in a sample, but without the sample simplification or complex-specific resolution provided by prior SDS-PAGE or native PAGE.

MS instrumentation, as well as membrane-protein sample preparation (Whitelegge, 2013; Battchikova et al., 2015; Heinz et al., 2016) and bioinformatics capabilities, has improved over the last two decades to facilitate PSII life-cycle research (Table 2 summarizes the kinds of experiments that have been performed and the main MS instrumentation and features that enable them). Early mass spectrometers that were applied to PSII research, especially triple-quadrupole (QqQ) and MALDI-time-of-flight (MALDI-TOF) instruments, had relatively low sensitivity, resolving power, and mass accuracy (on the order of 100-several hundred ppm; Michel et al., 1988; Sharma et al., 1997a,b,c; Frankel et al., 1999). Scarcity of genomic sequence data combined with low instrument sensitivity, mass accuracy, and fragmentation efficiency meant that sample analysis was mainly restricted to highly purified PSII complexes or individual subunits, with poor capability for novel protein identification. The mid-2000s saw the appearance of higher-performing instruments, especially the hybrid quadrupole-TOF (Q-TOF) and increasing availability of genomic sequence data for commonly studied photosynthetic organisms. These enabled routine bottom-up identification of the main subunits of PSII complexes (those with soluble domains) from more complex starting mixtures and identification of novel PSII-associated proteins (Kashino et al., 2002; Heinemeyer et al., 2004; Komenda et al., 2005). The fragmentation efficiency of the Q-TOF, however, still limited sequence coverage of proteins. The development and distribution of Fourier transform instruments (ion cyclotron resonance and orbitraps) sometimes interfaced with ion traps provided improved fragmentation efficiency and enabled analysis of highly complex mixtures with higher sequence coverage than ever before. These instruments allow proteome-wide experiments, enable routine confident PTM site identification, and have opened the door for bottom-up MS experiments on photosynthetic systems not before feasible (see Table 2 and sections below).

**Table 2:** **MS instruments and instrument features for PSII life-cycle research applications**.

**Biological application**	**Mass accuracy (MS^1^)**	**Mass accuracy (MS^2^)**	**Sensitivity/Good sequence coverage**	**QqQ**	**TOF**	**Q-TOF**	**LTQ-Orbitrap**	**Q-Exactive^a^**	**LTQ-FT-ICR**
ID proteins-purified PSII complex/simple mixture/gel band	Med	Low	Low	+	+	++	++	++	++
ID LMM subunits-purified PSII complex-intact/top-down	Med	Low	Low	+	+	++	++	++	++
ID proteins-membranes/complex mixture/unknown protein search	High	Med	Med	−	−	+	++	++	++
ID modifications-targeted search	High	Med	Med	+	+	+	++	++	++
ID modifications-non-targeted search (PTMs, footprinting)	High	Med	High	−	−	−	+	++	+
Quantification of proteins/modifications^b^-targeted search	High	Med	Med	+	+	+	++	++	++
Quantification of proteins/modifications^b^-non-targeted search	High	Med	High	−	−^c^	−	+	++	+
Cross-linking	High	Med-High	High	−	−	−	+	++	+

*High: high priority; Med: medium priority; Low: low priority. “−,” undesirable instrument choice; “+,” acceptable instrument choice; “++,” desirable instrument choice*.

a *The Q-Exactive is the most sensitive instrument listed. For experiments where it is given an equal rating as other instruments, high sensitivity was not deemed absolutely critical to the experiment. However, if a Q-Exactive is readily accessible, it is generally the preferred choice of the instruments listed. Other high-performing instruments have been released recently and are expected to be highly useful for PSII research as well*.

b *Ratings are assuming precursor-ion-based quantification, as has been used in the large majority of studies focused on the PSII life-cycle. Product-ion-based quantification is relevant for studies that use iTRAQ and some forms of spectral counting*.

c *As an exception, rough quantification of relative LMM subunit stoichiometry between samples has been performed by intact-mass measurement on a MALDI-TOF (Sugiura et al., 2010a)*.

#### The low-molecular-mass (LMM) subunits

Fully assembled PSII contains around 13 low-molecular-mass (LMM) proteins (< 10 kDa) whose transmembrane domains account for around 40–85% of the sequence. Identification of these very hydrophobic proteins by bottom-up LC-MS/MS is challenging, with typically four or fewer LMM proteins detected (Granvogl et al., 2008; Haniewicz et al., 2013; Pagliano et al., 2014). Difficulties are associated with the proteins' hydrophobicity and lack of soluble domains, which lead to sample losses during preparation, poor tryptic digestion due to infrequent arginines and lysines, slow elution during chromatography, and poor ionization efficiency due to lack of abundant proton-accepting residues. Fractionation by gel electrophoresis carries the additional challenge of extracting the protein from the gel, made more difficult because tryptic digestion sites are infrequent (Granvogl et al., 2008).

To circumvent these difficulties, intact-mass measurement (no MS/MS fragmentation of the protein) and more recently top-down MS strategies have been employed, both of which avoid protein digestion and are able to identify nearly all the LMM subunits in a purified complex (summarized in Table 3). Intact-mass measurement of the LMM subunits was demonstrated by both ESI and MALDI methods, using QqQ and MALDI-TOF instruments (see references cited in Table 3). Both methods achieve roughly 50–200 ppm mass accuracy; especially without fragmentation data, this would typically not be enough for confident identification of an unknown protein. However, because there are only approximately 13 LMM subunits, predicted masses, which are available from genomic sequences in many organisms, are distinctive, and because the starting sample is typically a purified PSII complex, these intact-mass measurements are routinely accepted as confident identifications.

**Table 3:** **Identification of LMM subunits by MS**.

**MS technique**	**Ionization method**	**Number of LMM subunits identified**	**Instrument**	**Mass accuracy (MS^1^)**	**References**
Bottom-up	ESI, MALDI	0–4	Variety	~1–100 ppm (peptides)	Many, e.g., Kereïche et al., 2008; Plöscher et al., 2011; Haniewicz et al., 2013; Pagliano et al., 2014
Intact	ESI	9–11	QqQ	~50–200 ppm	Sharma et al., 1997a; Gómez et al., 2002; Laganowsky et al., 2009; Thangaraj et al., 2010
	MALDI	9–13	MALDI-TOF	~50–200 ppm	Sugiura et al., 2010a; Pagliano et al., 2011; Nowaczyk et al., 2012; Nakamori et al., 2014; Pagliano et al., 2014
Top-down	ESI	8–13	Q-TOF, LTQ-FTICR	3–30 ppm (Q-TOF), < 1–5 ppm (LTQ-FTICR)	Granvogl et al., 2008; Plöscher et al., 2009; Thangaraj et al., 2010; Boehm et al., 2011, 2012
	MALDI	5	MALDI-TOF/TOF	~50–200 ppm	Pagliano et al., 2014

MS/MS fragmentation of intact LMM subunits, however, can be induced using both ESI and MALDI, although ESI has been more successful (see Table 3 and references cited therein). Whitelegge and co-workers (Thangaraj et al., 2010) identified 11 LMM proteins in purified PSII from *G. sulphuraria* with a linear ion trap quadrupole-Fourier transform ion cyclotron resonance (LTQ-FTICR) instrument after offline LC and confirmed several modifications. They employed both collisional-activated dissociation (CAD) and electron-capture dissociation (ECD) to fragment the proteins, but CAD gave better results for all LMM proteins. Eichacker and co-workers (Granvogl et al., 2008) demonstrated top-down analysis on a Q-TOF with sequence coverage ranging from 14 to 82%. This method has been used in several other recent studies (Plöscher et al., 2009; Boehm et al., 2011, 2012). Notably, Eichacker and co-workers (Granvogl et al., 2008) developed a protocol to perform in-gel extraction of intact LMM proteins prior to top-down analysis (capable of extracting all but the PsbZ protein from the gel matrix). This technique can be used to analyze individual BN-PAGE bands and, thus, identify the LMM components specific to individual types of PSII complexes in heterogeneous mixtures such as a thylakoid membrane proteome or affinity-tagged PSII complexes.

### PSII life-cycle application: Composition of subcomplexes

**Figure 2:**
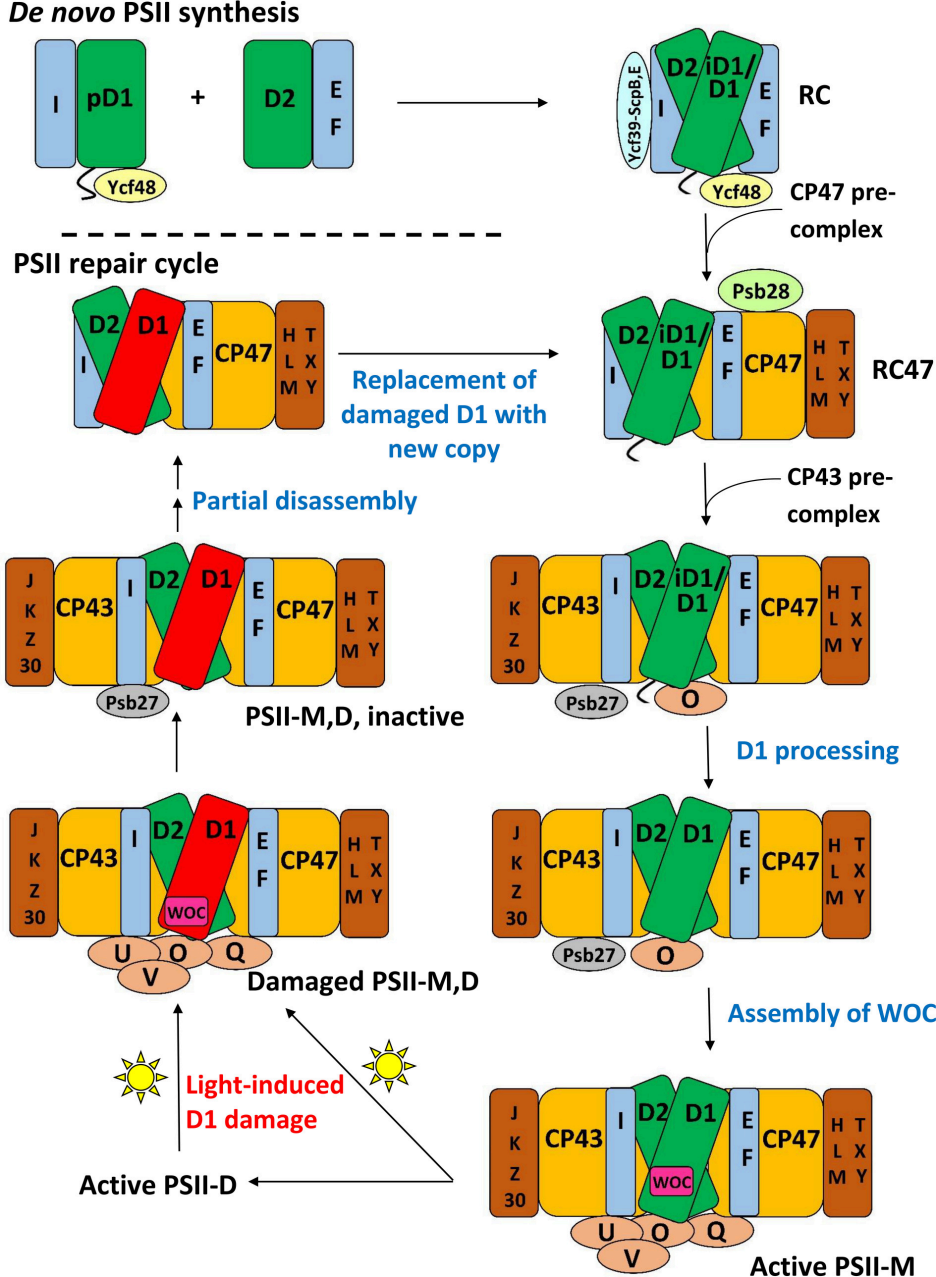
**A schematic of the PSII life-cycle**. Refer to the text for description of each step. This schematic represents the cyanobacterial PSII life-cycle. The subcomplex progression is similar in algae and higher plants, though several homologous subunits are named differently in these species than in cyanobacteria, and certain subunits are unique to each group (see Rokka et al., 2005; Shi et al., 2012; Nickelsen and Rengstl, 2013; Järvi et al., 2015; Heinz et al., 2016). In algae and higher plants, damaged complexes migrate from thylakoid grana to stromal lamellae for repair and the first steps of reassembly (Tikkanen and Aro, 2014; Järvi et al., 2015). In cyanobacteria, chloroplasts and such inter-thylakoid structure are absent, and repair is not believed to require spatial migration of damaged complexes. *De novo* PSII synthesis through RC formation appears to begin in specialized membrane subfractions in cyanobacteria, algae, and higher plants before PSII migration to the general thylakoid membrane space, though the details of this process in the various species classes remains to be resolved (Zak et al., 2001; Nickelsen et al., 2011; Nickelsen and Rengstl, 2013; Rast et al., 2015). E, F, H, I, J, K, L, M, O, Q, T, U, V, X, Y, Z, and 30 refer to the PsbE, PsbF, PsbH, etc. proteins, respectively. PSII-M, PSII monomer; PSII-D, PSII dimer.

Many subcomplexes form during the PSII life-cycle, and MS has played a critical role, in combination with gel electrophoresis, immunoblotting, crystallography, electron microscopy and other biochemical techniques, in identifying their components (Heinz et al., 2016). A schematic of the life-cycle is shown in Figure 2 (for reviews of the life-cycle and the subcomplexes that form, see Baena-González and Aro, 2002; Aro et al., 2005; Nixon et al., 2010; Shi et al., 2012; Komenda et al., 2012b; Nickelsen and Rengstl, 2013; Järvi et al., 2015; Heinz et al., 2016). A summary of the main subcomplexes whose composition has been studied by MS is found in Table 4 (for completeness, several other subcomplexes are also included). MS analysis generally allows more rapid, comprehensive, and definitive profiling of PSII subunits than other methods, and is especially useful for the LMM subunits that tend to stain poorly on gels. However, owing to the high sensitivity of MS and because relative quantification by MS is not straightforward, it can be difficult to distinguish a trace component of a complex from one that is stoichiometric. Immunoblotting, therefore, complements MS for characterizing composition of subcomplexes.

**Table 4:** **Composition of complexes in the PSII life-cycle by MS and other methods**.

**Sub-complex**	**Composition by MS**	**Species^a^**	**References**	**Composition by other methods**	**Species^a^**	**References**
D1 pre-complex	ND	–	–	pD1, I, (Ycf48^c^)	*Syn*. 6803	Dobáková et al., 2007
D2 pre-complex	ND	–	–	D2, E, F	*Syn*. 6803	Komenda et al., 2008
CP47 pre-complex	CP47, H, L, T	*Syn*. 6803	Boehm et al., 2011	CP47, H	*Syn*. 6803	Komenda et al., 2005
CP43 pre-complex	CP43, K, Psb30	*Syn*. 6803	Boehm et al., 2011	CP43, K, Z, Psb30^c^	*C. reinhardtii, T. elongatus*	Sugimoto and Takahashi, 2003; Iwai et al., 2007
RC	D1, D2, E, F, I	Pea	Sharma et al., 1997a,b	D1, D2, E, F, I, (W^d^)	Spinach	Nanba and Satoh, 1987; Ikeuchi and Inoue, 1988; Irrgang et al., 1995
RCII^*^	D1/iD1, D2, E, F, I, Ycf48, Ycf39, ScpB, ScpE	*Syn*. 6803	Knoppová et al., 2014	D1/iD1, D2, E, F, I, Ycf48, Ycf39, ScpE	*Syn*. 6803	Komenda et al., 2004; Dobáková et al., 2007; Komenda et al., 2008; Knoppová et al., 2014
RCIIa	ND	–	–	D1/iD1, D2, E, F, I, Ycf48	*Syn*. 6803	Dobáková et al., 2007; Komenda et al., 2008; Knoppová et al., 2014
RC47 monomer	D1, D2, CP47, E, F, I, L, M, T, X, Y, Psb28, Psb28-2	*Syn*. 6803	Boehm et al., 2012	D1, D2, CP47, E, F, H, Psb28, Psb28-2	*Syn*. 6803	Komenda et al., 2004; Dobáková et al., 2009; Boehm et al., 2012
RC47 monomer	D1, D2, CP47, E, F, I, T_c_, W	Spinach	Zheleva et al., 1998	D1, D2, CP47, E, F, H, I, M, R, T_c_	Spinach	Rokka et al., 2005
RC47 dimer	D1, D2, CP47, E, F, I, K, L, T_c_, W	Spinach	Zheleva et al., 1998	ND	–	–
PSII monomer, inactive	Psb27	*Syn*. 6803, *T. elongatus*	Grasse et al., 2011; Liu et al., 2013a	RC47 components + CP43, Psb27	*Syn*. 6803, *T. elongatus*	Roose and Pakrasi, 2008; Grasse et al., 2011; Liu et al., 2013a
PSII monomer/dimer, active	D1, D2, CP47, CP43, E, F, H, I, J, K, L, M, O, Q, U, V, T, X, Y, Z, Psb30	*Syn*. 6803, *T. elongatus*	Kashino et al., 2002; Nowaczyk et al., 2012	Crystal structure shows all components as by MS except PsbQ	*Syn*. 6803, *T. elongatus*	Roose et al., 2007; Umena et al., 2011
PSII monomer/dimer, active	D1, D2, CP47, CP43, E, F, H, I, K, L, M, O, R, T_C_, X	*N. tabacum*	Granvogl et al., 2008; Haniewicz et al., 2015	D1, D2, CP47, CP43, O, P, S; R, W (pea)	*A. thaliana*	Caffarri et al., 2009; Pagliano et al., 2011
PSII-LHCII supercomplexes^b^	D1, D2, CP47, CP43, E, F, H, I, K, L, M, O,R, T_C_, W, X; Lhcb1-4, 6	*N. tabacum*	Granvogl et al., 2008; Haniewicz et al., 2015	D1, D2, CP47, CP43, O, P, Q, S; Lhcb1-6; W (pea)	*A. thaliana*	Thidholm et al., 2002; Caffarri et al., 2009; Kouřil et al., 2012
PSII-PSI-PBS megacomplex	D1, D2, CP47, CP43, O, U, V; PsaA, B, C, D, E, F, L, Slr0172, Ycf4, PC; ApcA, B, C, D, E, F, CpcA, B, D, G1, G2	*Syn*. 6803	Liu et al., 2013b	ND	–	–
PSII-PSI-LHCII megacomplex	D1, D2, CP47, CP43, E; PsaA,B,L; LhcbM1, LhcbM10, Lhca2	*Ulva* sp.	Gao et al., 2015	D1, D2, CP47, CP43; PsaB,D,F,G, K,L; unspecified LHCII subunits	*A. thaliana*	Järvi et al., 2011

*ND, not determined*.

a *When two species are listed in the same subcomplex entry, the protein components are the union of those found in the individual studies*.

b *Characterization of specific PSII-LHCII supercomplexes*.

c *Uncertain; evidence is suggestive*.

d *Subsequent studies indicate PsbW presence in this complex may be an artifact of solubilization conditions (discussed in the text)*.

At the start of *de novo* PSII assembly, each of the four core subunits D1, D2, CP47, and CP43, forms a pre-complex with specific LMM components. Using a ΔD1 mutant in *Synechocystis* sp. PCC 6803 (hereafter *Synechocystis* 6803) and top-down ESI-MS on a Q-TOF, Nixon and co-workers (Boehm et al., 2011) showed that the CP47 pre-complex contains the LMM subunits PsbH, PsbL, and PsbT, whereas the CP43 pre-complex contains the LMM subunits PsbK and Psb30. In this study, it was not possible by MS alone to demonstrate fully stoichiometric binding, just co-purification, of those LMM subunits to CP47 and CP43. However, these results are consistent with the PSII crystal structures and other non-MS-based results (Boehm et al., 2011 and references cited therein). Previous evidence implies PsbZ could also associate with the CP43 pre-complex (Iwai et al., 2007; Guskov et al., 2009; Takasaka et al., 2010), but it was not detected by MS in this study. As determined by affinity purification and immunoblotting, the D1 pre-complex contains PsbI and possibly Ycf48 (Dobáková et al., 2007). It was suggested that a Ycf39-ScpB-ScpE complex may also associate as early as this stage to insert chlorophyll into D1 (Knoppová et al., 2014). The D2 pre-complex contains PsbE and PsbF (Müller and Eichacker, 1999; Komenda et al., 2008).

The D1 and D2 pre-complexes merge to form the reaction center (RC) complex, the earliest subcomplex capable of charge separation (Baena-González and Aro, 2002; Dobáková et al., 2007). The RC complex, initially isolated from spinach and wheat by detergent solubilization of thylakoid membranes, was characterized by gel electrophoresis and immunoblotting to contain D1, D2, PsbE, PsbF, and PsbI (Nanba and Satoh, 1987; Ikeuchi and Inoue, 1988). Intact-mass and bottom-up MS studies later confirmed this composition (Sharma et al., 1997a,b,c). Several biochemical studies detected the 10-kDa PsbW subunit, which is found in green algae and higher plants but not in cyanobacteria, as an additional component (Irrgang et al., 1995; Lorković et al., 1995; Shi and Schröder, 1997). Subsequently, more specific studies (including an MS-based one, Granvogl et al., 2008) showed that PsbW associates later, to dimers during formation of PSII-Light-harvesting complex II (LHCII) supercomplexes (see below; Shi et al., 2000; Thidholm et al., 2002; Rokka et al., 2005; Granvogl et al., 2008). Despite attaching to PSII at a late stage of assembly, PsbW may bind tightly to the D1/D2 surface and, thus, remain partially attached to the RC complex during solubilization, while other peripheral PSII subunits are removed, explaining the controversy (Rokka et al., 2005). This case highlights that subcomplexes obtained from detergent solubilization, a technique used especially in early PSII subcomplex studies, do not necessarily represent subcomplexes that form *in vivo*. An alternative major method for isolating PSII subcomplexes is purifying them from mutant strains that are “blocked” at a particular stage of assembly. Such complexes are indeed formed *in vivo*, but it is possible that the altered relative quantity of PSII subunits in the thylakoid membrane arising from the mutation may lead to artefactual binding of certain subunits to some subcomplexes (Thidholm et al., 2002). In cyanobacteria, two slightly different forms of the RC complex were observed, labeled RCII^*^ and RCIIa, which differ slightly in accessory protein content (see Table 3 and the section below). MS was critical in RCII^*^ component characterization, and was indirectly used for RCIIa characterization as well by gel and immunoblot comparison (Knoppová et al., 2014).

The next complex formed during PSII assembly is the RC47 intermediate, also called the CP43-less core monomer in plants, formed by attachment of the CP47 pre-complex to the RC complex. In 1998, Barber and co-workers (Zheleva et al., 1998) showed by MS that the monomeric RC47 complex from spinach contains the D1, D2, CP47, PsbE, PsbF, PsbI, PsbT_c_, and PsbW proteins, and the dimeric form contains, in addition, PsbK and PsbL. From the later studies on PsbW cited above, PsbW presence may arise from a tight binding to the D1/D2 surface, not *in vivo* presence in the RC47 complex during assembly. Based on Nixon and co-workers' study (Boehm et al., 2011) on the CP47 pre-complex in *Synechocystis* 6803, it would be expected that RC47 also contains PsbH. Indeed, a more recent MS-based study of the RC47 complex from *Synechocystis* 6803 identified all the proteins found by Barber and co-workers (Zheleva et al., 1998) in their monomeric RC47 complex (except PsbW which is not found in cyanobacteria), plus PsbH, PsbM, PsbX, PsbY, and Psb28 (Boehm et al., 2012).

Attachment of the CP43 pre-complex to RC47 forms the inactive PSII monomer (Nickelsen and Rengstl, 2013). Active monomeric PSII is formed upon D1 processing (Liu et al., 2013a), dissociation of Psb27 (Liu et al., 2013a), assembly of the water-oxidizing manganese-calcium cluster and photoactivation (Dasgupta et al., 2008), and binding of PsbO, PsbU, and PsbV (cyanobacteria) or PsbO, PsbP, and PsbQ (algae and higher plants; Bricker et al., 2012). Active monomers dimerize and can attach to the phycobilisome antenna complex (cyanobacteria) (Mullineaux, 2008) or various oligomeric states of LHCII complexes (algae and higher plants) (Kouřil et al., 2012).

Although, crystal structures of active PSII dimers from cyanobacteria are available, several MS studies of fully-assembled cyanobacterial PSII have provided independent confirmation of the subunits present in purified complexes under more native conditions (Sugiura et al., 2010a; Nowaczyk et al., 2012). Using native conditions has even helped discover a component (PsbQ) that was lost during crystallization (Kashino et al., 2002; Roose et al., 2007). The majority of PSII from algae and higher plants is found in several PSII dimer-LHCII supercomplexes (for a review see Kouřil et al., 2012). MS studies (in concert with other techniques) have identified their subunit compositions, even in the absence of crystal structures of the complexes from these organisms. Eichacker and co-workers (Granvogl et al., 2008) showed that the four PSII-LHCII supercomplexes in *Nicotiana tabacum* contain identical PSII core and LMM subunits (of the eight LMM subunits identified), and that only PSII-LHCII supercomplexes contain the PsbW protein. These results support previous studies that suggest that PsbW may facilitate linkage of LHCII trimers to PSII (Shi et al., 2000; Thidholm et al., 2002; Rokka et al., 2005). Using both bottom-up and top-down MS techniques, Pagliano et al. (2014) found that the various supercomplexes in pea contain identical core and LMM subunits, but that the C_2_S_2_M_2_ supercomplex contains the PsbQ, PsbR, PsbP, Lhcb3, and Lhcb6 proteins whereas the C_2_S_2_ supercomplex does not. In light of the stabilizing effect of the PsbQ and PsbP proteins on oxygen evolution, this finding raises interesting questions about the role of the C_2_S_2_ supercomplex. Another recent study used MS to characterize PSII-LHCII supercomplexes in *N. tabacum* and found a few differences in subunit composition; in particular, the C_2_S_2_ supercomplex contained Lhcb1 isoform CB25, while the C_2_S_2_M_2_ supercomplex did not (Haniewicz et al., 2015).

Several studies indicate that PSII-PSI-antenna megacomplexes can form in both cyanobacteria and higher plants. Using *in vivo* cross-linking, Blankenship and co-workers (Liu et al., 2013b) captured a PSII-PSI-phycobilisome megacomplex in *Synechocystis* 6803. The authors used MS to demonstrate presence of subunits from each complex in the preparation (Tables 3, 5), and identified cross-links revealing specific inter-complex subunit interactions. Aro and co-workers (Tikkanen et al., 2008b, 2010) showed that LHCII can transfer excitation energy to PSI in grana margins of higher plants as a means of balancing energy flux under varying light conditions. In support of this hypothesis, two PSII-PSI-LHCII megacomplexes from *Arabidopsis thaliana* were observed by a novel large-pore BN-PAGE system (Järvi et al., 2011), and more recently, a PSII-PSI-LHCII megacomplex was identified by MS from the macroalga *Ulva* sp. under drought stress conditions (Gao et al., 2015).

**Table 5:** **Summary of MS-based PSII cross-linking studies^a^**.

**Species**	**Cross-linked subunit 1**	**Cross-linked subunit 2**	**Cross-linker**	**Method notes**	**References**
*Syn*. 6803	Psb27	CP43	EDC, DTSSP	• Cross-linked species enriched on gel	Liu et al., 2011a
				• In-gel digestion; trypsin or chymotrypsin	
				• LTQ-Orbitrap XL	
				• MassMatrix search software	
*Syn*. 6803	PsbQ	CP47, PsbO	EDC, DTSSP	• No cross-link enrichment	Liu et al., 2014b
				• In-solution digestion; trypsin	
				• LTQ-Orbitrap XL	
				• MassMatrix search software	
*Syn*. 6803	D2, CP43, CP47	ApcE	DSP	• No pre-MS cross-link enrichment	Liu et al., 2013b
				• In-solution digestion; trypsin + LysC	
				• LTQ-Orbitrap XL	
				•≥ +3 charge states selected for MS^2^ to maximize cross-link selection	
				• MassMatrix search software	
*T. elongatus*	Psb27	CP43	BS^3^ d0/d12	• Isotope-encoded cross-linker	Cormann et al., 2016
				• No cross-link enrichment	
				• In-solution digestions; trypsin	
				• Orbitrap Elite Velos Pro	
				• StavroX search software	
*C. reinhardtii*	PsbP	PsbQ	EDC	• Wash step isolates extrinsic proteins after cross-linking	Nagao et al., 2010
				• Cross-linked species enriched on gel	
				• In-gel digestion; trypsin or Asp-N	
				• Ultraflex MALDI-TOF	
				• MS^1^ only; trypsin and Asp-N samples independently indicate the same cross-linked residues	
Spinach	PsbP	PsbQ	BS^3^	• Wash step isolates extrinsic proteins after cross-linking	Mummadisetti et al., 2014
				• Cross-linked species enriched on gel	
				• In-gel digestion; trypsin ± LysC	
				• LTQ-FTICR	
				• MassMatrix search software	
Spinach	PsbP	PsbE	EDC	• Biotin-tagged PsbP isolates the free protein + its cross-linked partners	Ido et al., 2012
				• Cross-linked species enriched on gel	
				• In-gel digestion; trypsin	
				• LTQ-Orbitrap XL	
				• MassMatrix search software	
Spinach	PsbP	PsbR, CP26	EDC	• Biotin-tagged PsbP isolates the free protein + its cross-linked partners	Ido et al., 2014
				• Cross-linked species enriched on gel	
				• In-gel digestion; trypsin	
				• LTQ-Orbitrap XL	
				• MassMatrix search software	

a *Only inter-protein cross-links that reveal interactions not detectable in the available PSII crystal structures are shown here*.

### PSII life-cycle application: Identification of accessory proteins

Many accessory proteins bind transiently to PSII subcomplexes during the PSII life-cycle, serving key regulatory roles, but are not present in the crystal structure owing to their absence in fully assembled PSII. For reviews of the accessory proteins of PSII (see Shi et al., 2012; Komenda et al., 2012b; Nickelsen and Rengstl, 2013; Mabbitt et al., 2014; Järvi et al., 2015; Heinz et al., 2016). Bottom-up MS has played a key role in identifying some of the known ones, and others likely remain to be identified. Identifying a previously unknown PSII-associated protein in this manner, however, is not straightforward because the mass spectrometers used for bottom-up analysis are so sensitive that dozens of contaminant proteins are often detected even in “purified” complexes. Low signal intensity of a peptide compared to those of known PSII peptides does not necessarily indicate a contaminant at low abundance because different peptides have different intrinsic ionization efficiencies, and many accessory proteins bind sub-stoichiometrically to PSII. Certain contaminant proteins such as NDH-1 complex subunits (Nowaczyk et al., 2012), ATP synthase subunits (Komenda et al., 2005), phycobilisome subunits (Kufryk et al., 2008), certain ribosomal proteins (Liu et al., 2011b), and several carbon dioxide-concentrating mechanism proteins (Kufryk et al., 2008; Liu et al., 2011b) are frequently observed. Careful examination of the full list and consideration of the experimental conditions are needed to distinguish plausible PSII-interaction candidates from contaminant proteins (Kashino et al., 2002). Although, different MS search software packages use different algorithms for scoring protein hits, a strict statistical confidence threshold should be employed and reported. Overall, although a simple bottom-up experiment is a powerful tool to suggest new candidate proteins that associate with PSII, subsequent targeted experiments on each one are needed to confirm the interaction.

This strategy has proven successful many times for identifying new PSII interaction partners. An early example (Kashino et al., 2002) analyzed SDS-PAGE bands by MALDI-TOF MS from a highly purified PSII preparation and identified several novel proteins, Sll1638 (PsbQ), Sll1252, and Sll1398 (Psb32), that appeared to be plausible PSII interaction partners. Follow-up biochemical studies targeting these proteins confirmed their role in the PSII life-cycle and elucidated functional aspects of each (Inoue-Kashino et al., 2011; Wegener et al., 2011; Bricker et al., 2012). A later proteomic study of purified PSII complexes revealed that the Slr0144-Slr-0152 proteins, all part of one operon, associate with PSII, leading to further characterization of their role in PSII assembly (Wegener et al., 2008). In other cases, specific subcomplexes were isolated before MS analysis and identification of accessory proteins. For example, analysis of a gel band from Δ*ctpA*-HT3-PSII revealed that the Psb27 protein binds specifically to a PSII subcomplex that accumulates before D1 processing (Roose and Pakrasi, 2004), initiating the studies that ultimately elucidated its role in PSII assembly (Nowaczyk et al., 2006; Roose and Pakrasi, 2008; Grasse et al., 2011; Liu et al., 2011a,b; Komenda et al., 2012a). MS analysis showed that the Ycf39, ScpB (HliC), and ScpE (HliD) proteins bind specifically to the RCII^*^ form of the reaction center complex, but not the related RCIIa form (Knoppová et al., 2014). The specific binding of the accessory proteins Psb28 (Dobáková et al., 2009; Boehm et al., 2012) and Psb28-2 (Boehm et al., 2012) to the RC47 complex, and of Ycf48 to RCII^*^ and RCIIa (Knoppová et al., 2014), was initially discovered by immunoblotting, but the proteins' presence was confirmed by MS, strengthening the finding.

### PSII life-cycle application: Identification of PTMs

#### Identification of processing events to form mature PSII proteins

The D1 protein is synthesized as a precursor protein (pD1) with a C-terminal extension that gets cleaved during PSII assembly (Takahashi et al., 1988). An early study using peptide sequencing showed that in spinach, cleavage occurs after Ala-344, removing nine C-terminal residues (Takahashi et al., 1988). Several years later, it was found that, in *Synechocystis* 6803, cleavage also occurs after Ala-344, removing 16 C-terminal residues (Nixon et al., 1992). In this study, peptide sequencing as well as fast atom bombardment (FAB)-MS (a predecessor for ESI and MALDI) were used to pinpoint this cleavage site. Ala-344 serves as a ligand for a Mn ion in the water oxidation cluster (Umena et al., 2011) so that without cleavage, PSII remains incapable of oxygen evolution (Roose and Pakrasi, 2004). The extension, thus, protects early assembly intermediates from harmful premature water oxidation activity. Interestingly, although D1 in higher plants is cleaved in a single step, cyanobacterial D1 is cleaved in two steps, and an intermediate D1 (iD1) is formed transiently (Inagaki et al., 2001). Although, the iD1 cleavage site remained unknown for two decades, in 2007, MS and biochemical evidence demonstrated that the CtpA protease cleaves after Ala-352 to form iD1, which is then cleaved again after Ala-344 to form mature D1 (Komenda et al., 2007). The significance of the two-step cleavage remains unknown, although iD1 may serve as a signal for transferring an early PSII assembly intermediate from the cytoplasmic to the thylakoid membrane (Komenda et al., 2007).

The CP43 protein also appears to be cleaved before, or during an early stage of, PSII assembly. Tandem MS analysis identified a CP43 peptide in spinach starting with a modified form of Thr-15 (Michel et al., 1988). Based on the genomic sequence, the preceding residue is a leucine, so this peptide would not be a predicted trypsin cleavage product. It was also found that the N-terminus of CP43 is blocked from analysis by Edman degradation, likely owing to N-terminal modification. Taken together, these results show that the first 14 residues of CP43 are cleaved, leaving Thr-15 as the mature protein's N-terminus (Michel et al., 1988). Subsequent studies identified the corresponding CP43 peptide in *A. thaliana* (Vener et al., 2001) and *Synechocystis* 6803 (Wegener et al., 2008), suggesting that this cleavage is conserved. Crystal structures of cyanobacterial PSII were not able to resolve the most N-terminal portion of CP43, so those structures do not address this question of CP43 cleavage (Loll et al., 2005; Umena et al., 2011).

Cyanobacterial Psb27, PsbQ, and PsbP have unusually hydrophobic properties for soluble lumen-localized proteins and contain a lipoprotein signal motif and conserved cysteine in their N-terminal regions (Thornton et al., 2004; Nowaczyk et al., 2006; Fagerlund and Eaton-Rye, 2011). This led to the suggestion that they are N-terminally lipid-modified and, thus, anchored to the lumenal surface of the thylakoid membrane. Using lipase treatment and MALDI-TOF MS, Rögner and co-workers (Nowaczyk et al., 2006) showed that Psb27 from *Thermosynechococcus elongatus* does indeed contain such a modification. Also using MALDI-TOF MS, Wada and co-workers (Ujihara et al., 2008) confirmed this finding with Psb27 from *Synechocystis* 6803 and also found that *Synechocystis* 6803 PsbQ, recombinantly expressed in *E. coli*, is also N-terminally lipid modified. Notably, this group developed a method to extract lipid-modified peptides from a gel matrix after in-gel digestion, enabling downstream MS analysis (Ujihara et al., 2008). During PSII assembly, it is important that Psb27 binds to the lumenal surface before the other extrinsic proteins (Liu et al., 2013a), and the lipid anchor may facilitate this sequence by keeping Psb27 in close proximity at all times. A similar role for the lipid anchor of PsbQ was proposed recently (Liu et al., 2015). A lipid modification on PsbP has not yet been demonstrated although strong suggestive evidence indicates its presence (Fagerlund and Eaton-Rye, 2011).

#### Identification of phosphorylation sites

In the early 1980s, phosphorylation of the four PSII subunits that later came to be known as D1, D2, CP43, and PsbH, was observed. These studies were conducted *in vivo* and *in vitro* using ^32^P labeling of whole cells and thylakoid membranes from *Chlamydomonas reinhardtii* and pea, with detection of phosphoproteins by autoradiography (Owens and Ohad, 1982, 1983; Steinback et al., 1982). Immunoblotting with antibodies that recognize phosphorylated residues was introduced later and became another popular detection method (Rintamäki et al., 1997). Neither of these methods, however, reveal the modified residue. This information was first obtained by gas-phase sequencing using Edman degradation, which demonstrated that the PsbH phosphorylation site is Thr-2, its N-terminus, in spinach (Michel and Bennett, 1987) and *C. reinhardtii* (Dedner et al., 1988). Since then, MS analysis has replaced Edman degradation and become the dominant method for phosphorylation-site determination, as it is higher-throughput, more definitive, more sensitive, and not limited by N-terminal blockage (e.g., acetylation). The main sites identified are presented below (for reviews, see Vener, 2007; Pesaresi et al., 2011; Puthiyaveetil and Kirchhoff, 2013).

Tandem MS demonstrated phosphorylation of D1-Thr-2, D2-Thr-2, and CP43-Thr-15, the mature proteins' N-termini, in spinach (Michel et al., 1988), *A. thaliana* (Vener et al., 2001), and *C. reinhardtii* (Turkina et al., 2006). Phosphorylation of CP43 was also observed at Thr-20, Thr-22, and Thr-346 in spinach (Rinalducci et al., 2006), and at Thr-346 and Ser-468 in *A. thaliana* (Sugiyama et al., 2008; Reiland et al., 2009). MS analysis showed that PsbH is phosphorylated at its N-terminus in *A. thaliana*, supporting the Edman degradation data from spinach and *C. reinhardtii*, and additionally demonstrated phosphorylation of Thr-4 (Vener et al., 2001). Intact-mass MS evidence also indicates double PsbH phosphorylation in spinach and pea (Gómez et al., 1998, 2002). More recently, phosphorylation of the extrinsic proteins PsbP, PsbQ, and PsbR was observed in phosphoproteomic studies of *A. thaliana* (Sugiyama et al., 2008; Lohrig et al., 2009; Reiland et al., 2009). Although, not discussed here, phosphorylation of LHCII is well-documented, and it regulates state transitions in green algae and higher plants (for reviews see Lemeille and Rochaix, 2010; Minagawa, 2011; Schönberg and Baginsky, 2012; Tikkanen and Aro, 2014; Tikhonov, 2015).

Phosphorylation of PSII subunits is not absolutely required for PSII repair (Bonardi et al., 2005) but assists in transferring damaged PSII complexes from the stacked thylakoid grana to stromal lamellae, where repair occurs. Phosphorylation appears to induce architectural changes in the stacked grana and increase membrane fluidity in such a way as to promote mobility of damaged PSII centers to the stromal lamellae for repair (Tikkanen et al., 2008a; Fristedt et al., 2009, 2010; Herbstová et al., 2012; Järvi et al., 2015). For many of the PSII phosphorylation sites, light intensity and/or other environmental conditions affect the phosphorylation extent, with implications for the functional significance of these modifications. MS analysis has played a critical role in these quantitative studies, and methodology for such measurements is discussed in the dynamics section below. For reviews that discuss the role of PSII phosphorylation (see Pesaresi et al., 2011; Mulo et al., 2012; Schönberg and Baginsky, 2012; Järvi et al., 2015).

PSII phosphorylation may not be needed in cyanobacteria owing to the lack of spatial organization of thylakoids (Mulo et al., 2012). However, a recent global proteomics study of the cyanobacterium *Synechococcus* sp. PCC 7002 (hereafter *Synechococcus* 7002) found that a portion of D1 copies are phosphorylated at their N-terminus, Thr-2 (Yang et al., 2014), as in higher plants. This finding opens the possibility for a role of phosphorylation in PSII turnover in cyanobacteria.

#### Identification of oxidative and other modifications

Light is necessary for PSII function, but even low light intensities can lead to PSII damage, particularly of the D1 protein. Damage triggers partial PSII disassembly, D1 degradation, insertion of a new D1 copy, and PSII re-assembly (Nickelsen and Rengstl, 2013). When the rate of damage exceeds that of repair, photosynthesis is inhibited, referred to as photoinhibition. Photodamage can be initiated in several ways, but a common result of each mechanism is production of highly oxidizing species (e.g., singlet O_2_, other reactive oxygen species (ROS), or radical PSII cofactors). These species rapidly oxidize PSII subunits, ultimately rendering the complex non-functional. For reviews of the photoinhibition process (see Barber and Andersson, 1992; Adir et al., 2003; Pospíšil, 2009; Allahverdiyeva and Aro, 2012; Tyystjärvi, 2013).

Although oxidative damage of PSII was long believed to be responsible for photoinhibition (Telfer et al., 1994), MS studies provided the first concrete evidence for specific oxidative modifications of PSII. Bottom-up MS analysis of the D1 and D2 subunits from pea PSII found up to three +16 oxidative modifications (each representing incorporation of an oxygen atom) on certain peptides (Sharma et al., 1997c). Interestingly, not all peptides were oxidized, but the oxidized ones were all located near the predicted D1 and D2 redox cofactor sites, supporting the idea that radical redox cofactors themselves, or ROS produced by reaction with them, cause oxidative damage to PSII. More recently, Bricker and co-workers (Frankel et al., 2012, 2013b) used tandem MS to identify oxidized residues on spinach D1, D2, and CP43 that are located near the Q_A_, Pheo_D1_, and manganese cluster sites, all reasonable sources of oxidizing species. Additionally, tryptophan oxidation products in spinach were identified on CP43-Trp-365 and D1-Trp-317, which are located near the manganese cluster (17 and 14 Å, respectively, in the crystal structure from *T. elongatus*; Anderson et al., 2002; Dreaden et al., 2011; Kasson et al., 2012). By monitoring the digested peptides' absorption at 350 nm, the authors found that these tryptophan oxidations are correlated with increased light intensity and decreased oxygen evolution. Other modifications to PSII subunits were also detected by MS (Gómez et al., 2002, 2003; Anderson et al., 2004; Rexroth et al., 2007; Sugiura et al., 2013). Notably, a recent global proteomics study of *Synechococcus* 7002 identified many new PSII PTMs (Yang et al., 2014), but the functional significance of these modifications remains to be determined.

## Dynamics: Quantitative or semi-quantitative changes in PSII proteins and PTMs

### MS-based methods to study PSII dynamics

Most MS-based quantification experiments seek the relative, not absolute quantity of a protein or PTM in one sample compared to another. We focus here on relative quantification methods because nearly all the work on PSII dynamics fell into that category.

#### Gel-based quantification

Perhaps the most basic MS-based semi-quantitative method is in-gel digestion at the same band in two different sample lanes, prompted by a significant staining-intensity difference between the two bands. This approach was used frequently when analyzing different purified PSII complexes (Liu et al., 2011b; Knoppová et al., 2014), yielding information about accessory proteins that bind specifically to certain subcomplexes. A proper loading control (typically equal chlorophyll) must be used to ensure a meaningful comparison. Multiple proteins are typically identified by MS in both bands, however, so it may not be immediately apparent which protein is the main component (Liu et al., 2011b). Confirmation may be necessary by western blotting or one of the more quantitative MS-based techniques described below.

The accuracy of gel-based quantification can be improved by introducing a second electrophoretic separation dimension before in-gel digestion and LC-MS/MS. Semi-quantitative two-dimensional denaturing gel electrophoresis (2DE) (distinct from 2D BN-PAGE described above), a popular technique especially in early proteomics studies, usually first separates proteins by size and then on the basis of pI (Rabilloud et al., 2010). The difference in staining intensity indicates the relative content of that protein in each sample. Because two proteins migrate less often together in two dimensions than in one, separation and quantification accuracy are improved. 2DE is useful for large-scale studies such as whole-cell or whole-organelle proteome profiling that require higher-resolution separation than a 1D gel provides. However, in recent years, 2DE has declined in popularity owing to its numerous drawbacks (reviewed in Rabilloud et al., 2010) and the improvements in other more versatile quantitative MS methods. Such large-scale proteomics studies have detected expression-level changes in several PSII proteins in response to a variety of stress conditions (e.g., Ingle et al., 2007; Aryal et al., 2011; Li et al., 2011; Guerreiro et al., 2014). However, insights into the PSII life-cycle have mainly emerged from more focused studies on purified PSII complexes.

#### Label-free quantification

Some MS-based relative quantification methods use a so-called label-free approach, but the better approach, when feasible, is to introduce a stable isotope into the sample. For label-free quantification, the samples to be compared are analyzed by LC-MS/MS separately. A variety of software tools can then be used to obtain an extracted ion chromatogram (EIC) of any peptide. The EIC displays the total intensity (peak area) of that peptide. Comparing the intensities of the same peptide from two different samples indicates the relative content of that peptide in those samples. Although the concept is simple, accurate label-free quantification depends on a number of factors: equal sample loading (on a relevant basis, e.g., chlorophyll concentration), reproducible LC runs, lack of ion suppression, and appropriate normalization during data analysis. For quantification of proteins, data from component peptides must be merged in a statistically sound way (Bantscheff et al., 2012; Nahnsen et al., 2013). Thorough mass spectral sampling of possible precursors—not as crucial in non-quantitative experiments—is necessary for accurate peak definition, but that typically diverts instrument time from obtaining product-ion spectra that give information for peptide identification and sequence coverage (Bantscheff et al., 2012). Various strategies have been designed to address this challenge (e.g., data-independent acquisition approaches such as MS^E^ (Silva et al., 2006; Grossmann et al., 2010) and “all-ion fragmentation” (Geiger et al., 2010) especially when combined with Ultra-Performance LC (UPLC) (Bantscheff et al., 2012). Label-free quantification by spectral counting, which involves comparing the total number of product-ion (MS/MS) spectra obtained for a given peptide or protein, is a common approach (Lundgren et al., 2010), although that has been used in fewer PSII-related studies (Fristedt and Vener, 2011; Stöckel et al., 2011). Label-free quantification of intact proteins is more direct than comparing peptides, but best applied for small proteins. Intact-mass spectra (MALDI and ESI) of the LMM PSII proteins indeed have been used in a number of instances for label-free quantification between states (Laganowsky et al., 2009; Sugiura et al., 2010a).

#### Isotope label-based quantification

The alternative to label-free quantification is introduction of a stable isotope label into one of the two samples being compared (certain methods also allow greater multiplexing, see below). In contrast to the label-free approach, the labeled and unlabeled samples (often called “heavy” and “light”) are mixed and analyzed in a single LC-MS/MS run. The mass spectra of the light and heavy peptide show two peaks shifted slightly in mass. Comparison of their peak areas, just as in label-free quantification, indicates the relative amount of that peptide in each sample (Bantscheff et al., 2012). Although, comparing peak areas from a single LC-MS/MS run eliminates the concerns of label-free LC reproducibility and ion suppression, labeling introduces additional sample preparation steps and often involves costly reagents.

Isotopic labeling (with ^2^H,^13^C, ^15^N, or ^18^O) of all proteins can be accomplished during cell growth (metabolic labeling), or by labeling a subset of proteins or peptides at various stages after cell lysis (chemical or enzymatic labeling). In the SILAC method (“stable isotope labeling by amino acids in cell culture”; reviewed in Chen et al., 2015), addition of labeled arginine or lysine to the growth medium results in incorporation of only the labeled form of that amino acid into all proteins. Hippler and co-workers (Naumann et al., 2007) used a SILAC-based method to measure changes in expression of PSII subunits and other proteins in *C. reinhardtii* under iron deficiency, and Jacobs and co-workers (Aryal et al., 2011) used this method to measure light-dark diurnal cycles in *Cyanothece* sp. ATCC 51142. A more common approach in PSII life-cycle research, however, has been ^15^N metabolic labeling (see “Measuring the temporal dynamics of life-cycle events using isotopic labeling” below), in which the growth medium is modified so that the only nitrogen source is a labeled salt such as potassium nitrate or ammonium chloride (Gouw et al., 2010).

Isotopic labeling at the peptide or protein level during downstream processing after cell lysis is an alternative to metabolic labeling. Tandem mass tags (TMT) (Thompson et al., 2003), isotope tags for relative and absolute quantification (iTRAQ) (Ross et al., 2004), enzymatic ^18^O labeling, and isotope-coded affinity tags (ICAT) can be used in proteomics experiments in photosynthetic organisms (Thelen and Peck, 2007). TMT and iTRAQ are related approaches that have become popular recently (Bantscheff et al., 2012). Both modify peptides with one of several possible isobaric tags that produce reporter ions during MS/MS fragmentation. Each sample is labeled with a different tag, but owing to the tags' isobaric nature, identical peptides from each sample are observed together chromatographically and as a single peak in a low-resolving power mass spectrum. Each tag, however, contains a unique reporter ion that appears as a distinct peak in the product-ion (MS/MS) spectra, and the ratio of these ions reveals the relative amounts of that peptide in each sample. The iTRAQ reagent modifies primary amines, and TMT tags are available that modify primary amines, thiols, or carbonyl groups. Advantages of these labeling approaches include the ability to multiplex up to 8 or 10 samples, greater than with metabolic and other chemical labeling methods, and the isobaric nature of the same peptide across all samples reduces both LC separation demands and MS data complexity (Bantscheff et al., 2012). Although, many proteomics studies on photosynthetic organisms have used these chemical labeling methods, most have not focused on PSII life-cycle issues (Thelen and Peck, 2007; Battchikova et al., 2015). Two relevant examples include the detection of elevated PsbO cysteine oxidation under DCMU and dark conditions (Guo et al., 2014), and intriguing evidence that PSII thermotolerance in *Synechocystis* 6803 may arise in part from antenna trimming and an increased rate of electron transfer to the cytochrome *b*_6_/*f* complex (Rowland et al., 2010).

### PSII life-cycle application: Measuring changes in phosphorylation levels

As mentioned in the composition section above, phosphorylation of PSII subunits affects membrane fluidity and inter-thylakoid dynamics, thus playing a role in facilitating PSII turnover in green algae and higher plants (see the reviews cited in that section for in-depth treatment of this topic). Many of the studies that have contributed to our current understanding of this process used MS quantification techniques to compare phosphorylation levels between samples and under different environmental conditions.

When using peak-area-based label-free quantification to determine the change in a modified peptide between samples, it is crucial that the peak area of the unmodified peptide be taken into account as well, to distinguish a true change in modification *extent* from simply an increased level of protein expression in one of the states. This method is demonstrated in a study of phosphorylation and nitration in *A. thaliana* grown under low and high light regimes (Galetskiy et al., 2011b). The authors first normalized each modified-peptide peak area in each sample to that of its unmodified counterpart and then compared the modified peptides' normalized peak area to each other. This method can reveal *fold-changes in modification extent* between the two states, but not the absolute percentage of that peptide that contains the modification (the “*modification stoichiometry*”).

To find the modification stoichiometry, it is necessary to know in addition the relative “flyability” (ionization efficiency) of the modified and unmodified peptides. Vierstra and co-workers (Vener et al., 2001) showed that the relative flyabilities of six synthetic phosphopeptides and their non-phosphorylated counterparts are nearly identical. Suggesting this as a general phenomenon for phosphorylated peptides, they estimated the modification stoichiometry for the phosphorylated peptides of D1, D2, CP43, PsbH, and an LHCII protein. In 2010, Vener and co-workers (Fristedt et al., 2010) calculated the actual relative flyability ratio for these PSII peptide pairs, and reported reliable modification stoichiometry for these proteins for the first time under the various conditions in their study. Interestingly, the flyability ratios were indeed close to 1 for each pair (ranging from 0.89 to 1.23), supporting the earlier suggestion that this may be the case for most phosphorylated/non-phosphorylated peptide pairs (Vener et al., 2001). Other studies have since used those flyability ratios to determine changes in the modification stoichiometry of those same phosphorylation sites under other growth conditions (Fristedt and Vener, 2011; Romanowska et al., 2012; Samol et al., 2012). Knowledge of modification stoichiometry under different conditions is quite valuable; it enabled, for example, a greater level of confidence and detail in the model proposed for how phosphorylation affects thylakoid membrane stacking than would have been possible with fold-change data alone (Fristedt et al., 2010).

Chemical isotopic labeling of peptides has also been applied fruitfully to the study of PSII phosphorylation. Immobilized metal-ion affnity chromatography (IMAC) is a standard protocol for enrichment of phosphopeptides, taking advantage of the interaction between phosphoryl groups and a Fe^3+^-agarose matrix (Andersson and Porath, 1986). Given that free carboxyl groups can also interact with the resin, it has become common to convert free carboxylates to methyl esters after digestion and prior to IMAC, to avoid this interaction (Ficarro et al., 2002). Vener and co-workers (Vainonen et al., 2005) modified this approach by using deuterated methanol (CD_3_) as the esterification reagent for one sample, and unlabeled methanol for a second sample to quantify by “isotope encoding.” After mixing the samples and analyzing by LC-MS/MS, the relative amount of each phosphorylated peptide in the two samples is quantified by comparison of their mass spectral peak areas. It should be noted that this approach does not reveal the modification stoichiometry of any phosphorylation site; rather the techniques described above still need to be performed to gain that information. Instead, as with other isotope-labeling strategies, it enables more confident and straightforward comparisons of the level of any given peptide between samples. This labeling method was used to study phosphorylation of PSII under a variety of conditions and genetic backgrounds (Vainonen et al., 2005; Lemeille et al., 2010; Fristedt and Vener, 2011; Samol et al., 2012).

### PSII life-cycle application: Measuring changes in oxidation levels

As discussed above, oxidation of PSII subunits is a well-documented phenomenon, and occurs, at least partially, from oxidizing species generated during the electron transfer reactions of PSII, especially under stress. However, relatively few studies have quantified changes in PSII subunit oxidation under different controlled conditions. Adamska and co-workers (Galetskiy et al., 2011a) used label-free quantification to compare oxidation and nitration (also associated with oxidative stress) levels of thylakoid membrane protein complexes from *A. thaliana* grown under low and high light. They found significantly more modified sites in PSII than in the PSI, cytochrome *b*_6_/*f*, and ATP synthase complexes. Interestingly, the modified D1, D2, and PsbO sites increased around 2-5-fold, whereas CP47, CP43, PsbE, and PsbR oxidation levels remained roughly constant. D1 and D2 bind most of the redox-active cofactors of PSII, so the increased oxidation especially of these two proteins is not surprising. Similarly, by measuring the increase in 350 nm absorption, Barry and co-workers (Dreaden et al., 2011; Kasson et al., 2012) found that two tryptophan oxidation products increase after exposure to high light, with a corresponding decrease in oxygen evolution activity. Adamska and co-workers (Galetskiy et al., 2011b) found that nitration levels in assembled PSII complexes decrease after exposure to high light, but increase in PSII subcomplexes. This may imply that once nitrated, PSII complexes are damaged and targeted for disassembly and repair.

### PSII life-cycle application: Measuring the temporal dynamics of life-cycle events using isotopic labeling

Measurement of PSII subunit lifetimes has focused mainly on D1, using immunodetection following addition of a protein-synthesis inhibitor or by radioisotope pulse-chase labeling with detection by autoradiography or phosphorimaging (Aro et al., 1993; Mullet and Christopher, 1994; Ohnishi and Murata, 2006). Recently, several studies used ^15^N labeling pulses and quantified the disappearance of unlabeled PSII subunits using MS. This method enables simultaneous detection of a larger number of PSII subunits and eliminates any concern of overlapping signal from proteins with similar electrophoretic mobility (Yao et al., 2012b). From surveying nine PSII subunits from *Synechocystis* 6803, Vermaas and co-workers (Yao et al., 2012a) found that protein half-lives range from 1.5 to 33 h in a PSI-less mutant grown under low light (4 μmol m^−2^s^−1^ photon flux). In WT *Synechocystis* 6803 grown under 75 μmol m^−2^s^−1^ photon flux, half-lives of D1, D2, CP47, and CP43 ranged from < 1 to 11 h (Yao et al., 2012b). In both studies, D1 exhibited the shortest half-life. These studies highlight the wide range in PSII subunit lifetime and the tight regulation of protein synthesis and PSII assembly that must occur to ensure constant proper stoichiometric availability of all subunits. Interestingly, the chlorophyll half-life was several times longer than that of the core chlorophyll-binding proteins, but the half-life was reduced in the absence of the small CAB-like proteins (SCPs), implying that SCPs play a role in chlorophyll recycling during PSII turnover (Yao et al., 2012a).

Rögner, Nowaczyk, and co-workers demonstrated an elegant application of ^15^N labeling by purifying several subcomplexes in the PSII life-cycle after a pulse with ^15^N (from ^15^NH_4_Cl). Comparing extents of incorporation of ^15^N in different subcomplexes (e.g., monitoring D1 and D2 peptides) reveals the subcomplexes' position in the PSII life-cycle. Using this method, the authors demonstrated that in *T. elongatus*, Psb27 binds to a monomeric subcomplex early in the PSII assembly process (Nowaczyk et al., 2006), and that Psb27 binds again during disassembly to inactive dimers (Grasse et al., 2011). This information fits well with the current understanding of Psb27 as a gatekeeper preventing manganese cluster assembly in immature complexes (Liu et al., 2013a; Mabbitt et al., 2014).

Cyanobacteria contain multiple versions of the *psbA* gene, and the resulting versions of the D1 protein have some different properties and are expressed preferentially under different environmental conditions (for reviews see Mulo et al., 2009; Sugiura and Boussac, 2014). For example, the *psbA1* gene product in *T. elongatus* is dominant under standard growth conditions, but expression of the *psbA3* gene product, which differs from the PsbA1 copy by ~21 residues, increases under high light conditions (Clarke et al., 1993; Kós et al., 2008; Mulo et al., 2009). Characterization of PSII from mutants that express only specific versions of the gene has shown differences in electron-transfer properties, with the implication that PsbA3 assists in photoprotection of PSII under light stress conditions (Sander et al., 2010; Sugiura et al., 2010b). D1-copy expression was mainly monitored on the transcript level (Golden et al., 1986; Komenda et al., 2000; Kós et al., 2008; Sugiura et al., 2010b). However, using ^15^N labeling and MS-based quantification, Rögner and co-workers showed that PsbA3 incorporation on the protein level could be monitored unambiguously in *T. elongatus* under high light conditions (Sander et al., 2010) and in the Δ*psbJ* mutant (Nowaczyk et al., 2012). Those studies used ^15^N-labeled PSII from a strain that only expresses the PsbA3 copy as a standard for 100% incorporation; relative peak area of the unlabeled PsbA3 peptides compared to this standard is a measure of the incorporation. Such definitive monitoring should allow further detailed studies of *psbA* gene incorporation dynamics.

Progress has also recently been made on the role of the PsbA4 D1 copy; an iTRAQ labeling study found elevated expression of PsbA4 in *Cyanothece* sp. PCC 7822 in the dark (Welkie et al., 2014), providing complementary evidence to that of Pakrasi and co-workers (Wegener et al., 2015) who found that PsbA4 incorporation into PSII renders the complex non-functional. PsbA4 replaces PsbA1 at night in cyanobacterial species that fix nitrogen during this time, protecting against even the trace levels of oxygen evolution that could occur and damage the nitrogenase enzyme (Wegener et al., 2015).

## Structure: Determining protein-protein interactions in PSII complexes

### MS-based methods to study PSII structure

X-ray crystallography remains the benchmark for determining the structure of protein complexes, but besides fully-assembled active PSII, many complexes that form during the PSII life-cycle are too transient and low in abundance to be easily amenable to crystallography. Valuable information about protein-protein interactions within PSII was obtained from immunogold labeling (Tsiotis et al., 1996; Promnares et al., 2006) and yeast two-hybrid assays (Schottkowski et al., 2009; Komenda et al., 2012a; Rengstl et al., 2013), but the former is primarily suitable for large PSII complexes (Dobáková et al., 2009), and the latter is time-consuming and low-throughput. Both provide relatively low-resolution structural information. Recently, advanced structural proteomics techniques bypass the limitations of the above techniques and offer higher-resolution structural data (although still lower than X-ray crystallography). Either chemical cross-linking or protein footprinting followed by MS detection of these modifications are enabled by MS instruments with high sensitivity, resolving power, and < 1–5 ppm mass accuracy on orbitrap- and FTICR-based instruments (Table 2). These methods allow not only identification of the binding partners of a specific protein but also a low-resolution mapping of the binding site.

#### Chemical cross-linking

Briefly, the chemical cross-linking technique (reviewed in Sinz, 2014) uses a small molecule with two functional groups on either end that can react with protein residues, separated by a spacer arm (typically less than 14 Å). Many types of cross-linkers are available (Paramelle et al., 2013). The ones most commonly used in PSII research (Bricker et al., 2015) can react with either the primary amine of a lysine and protein N-terminus (and under certain conditions, to a lesser extent with the hydroxyl group of a serine, threonine, or tyrosine, Mädler et al., 2009), or with the carboxylate of aspartate and glutamate side chains and protein C-termini. After both sides of the cross-linker react with neighboring proteins, digestion, LC-MS/MS, and specialized data analysis can identify cross-linked peptides. Inter-protein cross-linked peptides provide structural information about the complex because the two linked residues are constrained to the spacer arm-length distance from each other.

Cross-linking has been used for decades to study protein-protein interactions (Clegg and Hayes, 1974; Wetz and Habermehl, 1979; Walleczek et al., 1989; Back et al., 2003; Sinz, 2014), but its power was limited until modern MS instrumentation and the proteomics platform enabled high-throughput analysis and confident identification of linked peptides (Rappsilber, 2011). Identification of cross-linked peptides by MS is more challenging than for a typical protein digest, especially for large complexes, because the candidate peptide database increases roughly with the square of the number of peptides. As a result, false positives based on the mass spectrum are common even with high mass accuracy instruments, making high-quality product-ion spectra critical for a confident assignment. Despite powerful and constantly improving cross-link search algorithms (Rinner et al., 2008; Xu and Freitas, 2009; Petrotchenko and Borchers, 2010; Götze et al., 2012, 2015; Yang et al., 2012; Hoopmann et al., 2015), manual verification of the product-ion spectra of hits is highly recommended. Successful cross-linking requires high sequence coverage and high mass accuracy as is now practical with orbitrap- and FTICR-based instruments (Table 2).

Because cross-linked peptides give typically low-intensity signals compared to those of unlinked peptides, they are often not selected for fragmentation by the instrument's traditional “highest-abundance ion” selection criteria. Several strategies have been developed to improve cross-link selection and/or reduce false positives. They include various methods to enrich for cross-linked peptides before LC-MS/MS (Chu et al., 2006; Kang et al., 2009; Fritzsche et al., 2012; Leitner et al., 2012); use of isotope-coded linkers whose “fingerprint” increases confidence in an identification and can enable real-time guided selection of cross-links for fragmentation (Müller et al., 2001; Pearson et al., 2002; Seebacher et al., 2006; Petrotchenko et al., 2014); and MS-cleavable linkers that simplify data analysis by cleaving a cross-linked peptide into its component peptides before fragmentation (Kao et al., 2011; Petrotchenko et al., 2011; Weisbrod et al., 2013; Buncherd et al., 2014).

#### Protein footprinting

Protein footprinting is another MS-based structural technique that has been used to study PSII. Its principle is that a protein residue's solvent accessibility determines its susceptibility to modification by a reagent in the solution; residues buried in a protein-protein interface are less susceptible to modification than surface-exposed residues. These modifications are then detected by MS. Instruments with high sensitivity, resulting in high sequence coverage, are critical so that footprinting experiments yield maximal information (Table 2). A common approach is hydroxyl radical footprinting using the well-established technique of synchrotron radiolysis of water to generate the radicals (Takamoto and Chance, 2006; Wang and Chance, 2011). Fast photochemical oxidation of proteins (FPOP) is a more recent hydroxyl radical fooptrinting technique that uses a laser pulse to generate the radicals and can probe protein dynamics that occur on a faster timescale, down to microseconds (Gau et al., 2011). Hydroxyl radical footprinting can modify 14 of the 20 amino acid side chains (Wang and Chance, 2011). Another technique, glycine ethyl ester (GEE) labeling, adapts a long-standing method for modifying and cross-linking carboxylate groups in proteins (Hoare and Koshland, 1967; Swaisgood and Natake, 1973) for protein footprinting (Wen et al., 2009; Gau et al., 2011). It is easier to implement than hydroxyl radical footprinting, and data interpretation is simpler, but it can only probe changes on aspartate, glutamate, and protein C-termini.

### PSII life-cycle application: Cross-linking and footprinting to determine interactions among PSII subunits

Early cross-linking studies on PSII provided information about subunit connectivity before PSII crystal structures were available. Many studies focused on the lumenal extrinsic proteins (Enami et al., 1987; Bricker et al., 1988; Odom and Bricker, 1992; Han et al., 1994), which are more easily accessible to soluble cross-linkers, but interactions involving the transmembrane subunits can also be detected (Tomo et al., 1993; Seidler, 1996; Harrer et al., 1998). In the absence of the MS-based platforms currently available, gel electrophoresis and immunoblotting identify cross-linked products and their likely component proteins. Those methods are still helpful today as confirmation and when cross-linked peptides are not detected by MS (Hansson et al., 2007; Nagao et al., 2010; Liu et al., 2011a, 2014b), but MS provides much greater confidence in the identification and pinpoints the exact cross-linked residues. Notably, Satoh and co-workers (Enami et al., 1998) used FAB-MS to identify intramolecular cross-linked peptides in PsbO, and deduced the linked residues even without MS/MS capability.

Since these early studies, crystal structures have elucidated the connectivity between the components of active cyanobacterial PSII. As a result, more recent cross-linking studies have focused on accessory proteins that bind only to subcomplexes and/or that are not found in the crystal structures, though work has continued on the lumenal extrinsic PSII subunits from algae and higher plants, PsbP and PsbQ, which differ significantly from their cyanobacterial counterparts (Bricker et al., 2012; results are summarized in Table 5). Cross-linking-MS has also been recently applied to study interactions within the phycobilisome (Tal et al., 2014) and between the phycobilisome and the photoprotective orange carotenoid protein (OCP) (Zhang et al., 2014; Liu et al., 2016), reviewed in Bricker et al. (2015).

With complementary use of the cross-linkers EDC and DTSSP, Pakrasi and co-workers (Liu et al., 2011a) demonstrated that the accessory protein Psb27 binds on the lumenal surface of CP43. Because this interaction is transient and occurs in only a small fraction of PSII centers in the cell at a given time, the authors purified PSII complexes from the Δ*ctp*A mutant strain of *Synechocystis* 6803 that accumulates such complexes (Liu et al., 2011b), maximizing chances of capturing and observing Psb27 inter-protein cross-links. The two cross-linked species detected were used to map Psb27 onto the PSII crystal structure, showing how Psb27 accomplishes its role as a gatekeeper, protecting partially assembled PSII complexes from gaining premature harmful water oxidation activity (Roose and Pakrasi, 2008). Recently, Nowaczyk and co-workers (Cormann et al., 2016) identified a different cross-link between Psb27 and CP43 in *T. elongatus* using an isotope-encoded version of the BS^3^ cross-linker. Despite the different cyanobacterial species used in the two studies, and the different Psb27 residues that were cross-linked, both cross-links localize Psb27 to the same domain on CP43 (Liu et al., 2011a; Cormann et al., 2016).

Cyanobacterial PsbQ is a component of active PSII (Roose et al., 2007), but is not found in any of the crystal structures, presumably because it is destabilized under crystallization conditions. Pakrasi and co-workers (Liu et al., 2014b) again used EDC and DTSSP in parallel and detected a PsbQ-CP47 and two PsbQ-PsbO cross-links by MS. A PsbQ-PsbQ cross-link that appears to arise from two different copies of the protein was also detected. Taken together, these results position PsbQ along the lumenal PSII dimer interface, consistent with evidence that PsbQ stabilizes the PSII dimer (Liu et al., 2014b). In this study, in-solution digestion was used instead of in-gel digestion to avoid losses of large cross-linked peptides that are difficult to extract from the gel matrix.

Several recent studies have probed the binding sites of the higher plant lumenal extrinsic proteins PsbP and PsbQ, which help optimize Ca^2+^ and Cl^−^ binding properties at the oxygen-evolving center (Bricker et al., 2012). Ifuku and co-workers (Ido et al., 2012, 2014) identified cross-links in spinach PSII between PsbP and PsbE, PsbR, and CP26 by MS and provided MS-based evidence for PsbP-CP43, PsbQ-CP43 and PsbQ-CP26 cross-links. The suggestive evidence arose from MS identification of CP43 or CP26 in individual cross-linked gel bands after affinity pull-downs using biotin-tagged PsbP or PsbQ (Ido et al., 2014). Their binding model for PsbP is different than that proposed by Bricker and co-workers (Mummadisetti et al., 2014), who identified nine intra-protein cross-links between the N-terminal and C-terminal regions of spinach PsbP that constrain significantly its binding conformation. The authors also identified a PsbP-PsbQ cross-link, consistent with that observed in *C. reinhardtii* by Enami and co-workers (Nagao et al., 2010).

The PsbQ-CP43 interaction in spinach PSII suggested by Ifuku and co-workers (Ido et al., 2014) contrasts with the PsbQ-CP47 cross-link identified in *Synechocystis* 6803 by Pakrasi and co-workers (Liu et al., 2014b) and their evidence for a PsbQ-PsbQ interaction at the PSII dimer interface. Significant sequence differences between cyanobacterial and plant PsbQ may explain this discrepancy. Bricker and co-workers (Mummadisetti et al., 2014) also found cross-linking evidence for a PsbQ-PsbQ interaction in spinach that may require a position at the dimer interface, consistent with the Pakrasi group's results in *Synechocystis* 6803. However, they suggest that that interaction could in theory arise from an inter-PSII-dimer interaction, and, thus, the results could alternatively be consistent with the Ifuku group's positioning of spinach PsbQ near CP43. Interestingly, the recently published crystal structure of PSII from the eukaryotic red alga *Cyanidium caldarium* indeed shows PsbQ′ binding to the lumenal surface of CP43 (Ago et al., 2016). PsbQ′ shares relatively low sequence homology to green algal or higher plant PsbQ; and though PsbQ′ can functionally replace PsbQ at least partially in *C. reinhardtii*, it cannot bind to spinach PSII (Ohta et al., 2003). Therefore, the red algal PsbQ′-CP43 interaction supports Ifuku and co-workers' (Ido et al., 2014) similar conclusion in spinach, but at the same time it does not necessarily contradict the alternate PsbQ-CP47 interaction observed by the other groups in spinach and *Synechocystis* 6803. The recent characterization of an active PSII complex from *Synechocystis* 6803 with multiple copies of the PsbQ protein (Liu et al., 2015) hints at one possible reconciliation of these findings, if such a complex is present in other species as well. Despite some discrepancies, these results begin to elucidate the binding orientation of the higher plant lumenal extrinsic proteins, suggesting a mechanism for stabilization of PSII-LHCII supercomplexes (Ido et al., 2014), and paving the road for further structural studies.

Although the advanced techniques for improving cross-link identification described in the methods section above have largely not yet been applied to PSII studies (with the exception of the recent use of isotope-encoded BS^3^ by Nowaczyk and co-workers, Cormann et al., 2016), several other creative approaches have been used. Enami and co-workers (Nagao et al., 2010) improved identification confidence by detecting the same cross-linked residues in peptides from two separate digestion experiments, one with trypsin and one with Asp-N. Pakrasi and co-workers (Liu et al., 2011a) provided strong evidence, using the thiol-cleavable cross-linker DTSSP and 2D gel electrophoresis, that Psb27 and CP43 cross-link to each other, allowing targeted data analysis and providing higher confidence in the subsequent MS cross-link identification. Ifuku and co-workers (Ido et al., 2012, 2014) used a biotin-tagged PsbP or PsbQ to purify only those cross-linked proteins. Although this method is not as efficient as purifying only cross-linked peptides by means of a tagged linker, because following digestion many non-linked peptides from the tagged protein will be present, it does simplify sample complexity and focuses on cross-links containing a particular protein of interest. Notably, Blankenship and co-workers (Liu et al., 2013b) demonstrated that *in-vivo* cross-linking of thylakoid membrane complexes is possible and can capture interactions between protein complexes that are otherwise difficult to preserve after cell lysis. Using the membrane-permeable cross-linker DSP, they captured a PSII-PSI-phycobilisome megacomplex and identified five cross-links between PSII subunits and the PBS, and five between PSI subunits and the PBS, providing the first molecular-level description of the interface of these complexes.

Like cross-linking, protein footprinting is a technique that has long been used in PSII structural studies but that has become significantly more powerful in combination with modern MS. Early studies using N-hydroxysuccinimidobiotin (NHS-biotin) and other modification reagents investigated the binding site of higher plant PsbO to PSII. In the absence of MS detection, specific modification sites could either not be identified (Bricker et al., 1988) or were localized to particular protein domains by N-terminal sequencing of peptides (Frankel and Bricker, 1992). With the rise of protein MS in the mid-1990s, MALDI-TOF and FAB-MS were used to identify modified peptides; lack of MS/MS capability, however, produced lower-confidence peptide identification than is achievable today, and meant that specific modified residues could only be pinpointed in favorable cases (Frankel and Bricker, 1995; Miura et al., 1997; Frankel et al., 1999). Nonetheless, these pioneering footprinting studies demonstrated, e.g., that PsbO interacts with Loop E of CP47 (Frankel and Bricker, 1992), and that charged residues on the surface of PsbO are involved in its interaction with PSII (Miura et al., 1997; Frankel et al., 1999).

Recently, hydroxyl radical footprinting using synchrotron radiolysis of water was used to study the binding surfaces of spinach PsbP and PsbQ to PSII, with detection of modified residues by MS (Mummadisetti et al., 2014). The results reveal buried regions on the surface of these proteins that complement the authors' cross-linking data and suggest these proteins' binding interfaces to other PSII subunits. The data also confirm and elaborate on the binding region identified by this group in a previous study using NHS-biotin as footprinting reagent (Meades et al., 2005).

Although the above footprinting studies detected whether or not a residue was modified in a given state, it is also possible to analyze footprinting data quantitatively to detect a conformational change in a complex in two different states. The label-free approaches described above can be used to monitor the relative change in modification, normalized to the unmodified peptide, in different PSII complexes. The utility of this approach was demonstrated in a study of the role of Psb27 in PSII assembly (Liu et al., 2013a) using GEE labeling. The authors monitored the relative changes in aspartate and glutamate modification of three PSII complexes representing different stages of PSII assembly, not only extending previous information about the Psb27 binding site (Liu et al., 2011a; Komenda et al., 2012a), but also demonstrating a conformational change upon D1 processing that prompts Psb27 dissociation and permits assembly of the oxygen evolving complex (Liu et al., 2013a). Blankenship and co-workers have also used quantitative GEE labeling to detect a light-dependent conformational change in the OCP protein that appears to underlie its photoprotective function (Liu et al., 2014a). The recent implementation of isotopically-labeled GEE (iGEE) footprinting (Zhang et al., 2016) will streamline, and increase confidence in, quantitative comparisons of modification extent between states.

Hydroxyl radical footprinting has also been used to identify putative water and oxygen channels in PSII (Frankel et al., 2013a), a topic that has been explored previously through computational studies (Murray and Barber, 2007; Ho and Styring, 2008; Gabdulkhakov et al., 2009; Vassiliev et al., 2012). This study provides general experimental support for the existence of such channels, confirms specific channel identifications from computational work (Ho and Styring, 2008; Vassiliev et al., 2012), and proposes a previously unidentified putative oxygen/ROS exit channel (see Bricker et al., 2015 for a discussion of the MS-based and computational results).

## Future directions

MS technology and associated sample preparation techniques are evolving rapidly. Increasing sensitivity and speed of instruments for bottom-up proteomics allows better coverage of transmembrane PSII proteins; for example, coverage of the core D1, D2, CP47, and CP43 proteins is routinely ~50–85% on a Thermo Q-Exactive Plus instrument, whereas ~20–40% coverage was reported on LTQ-Orbitrap, LTQ-FTICR, and MALDI-TOF instruments (Aro et al., 2005; Frankel et al., 2012; Liu et al., 2013a,b). This increased coverage will mean that more PTMs and cross-linked peptides can be identified, and a larger portion of the PSII complex can be mapped by footprinting. PTM analysis, especially using quantitative techniques to compare complexes exposed to different conditions, may help elucidate signals (largely unknown in cyanobacteria) that trigger D1 degradation. The increasing availability of high-sensitivity instruments that can achieve high sequence coverage is enabling detailed quantitative and non-quantitative global proteomic studies. The new challenge is to reduce the large amounts of information becoming available into specific testable hypotheses for targeted follow-up studies.

Improvements at all stages of the cross-linking workflow are occurring, from linker design to linked-peptide enrichment and software analysis. Specifically, isotope-labeled and MS-cleavable linkers are powerful tools that are just beginning to be applied to PSII research. *In-vivo* cross-linking is a promising approach to detect transient or unstable interactions that are difficult to capture after cell lysis. Cross-linking may enable binding site identification for at least some of the approximately 30 accessory proteins now known or believed to associate with PSII during its life-cycle (Nickelsen and Rengstl, 2013; Järvi et al., 2015). Detecting interactions between PSII subcomplexes and, e.g., D1 degradation proteases or proteins involved in chlorophyll loading would also be of prime interest.

Intact-mass measurements of the large core PSII proteins D1, D2, CP47, and CP43 were reported in several studies (Sharma et al., 1997b; Whitelegge et al., 1998; Huber et al., 2004; Thangaraj et al., 2010), with detection of the phosphorylated form of D1 as well in some cases (Whitelegge et al., 1998; Huber et al., 2004). However, their top-down analysis has not yet been achieved. Top-down technology is continuously developing, especially methods for increased product-ion sequence coverage (Frese et al., 2012; Shaw et al., 2013; Brunner et al., 2015) and analysis of larger integral membrane proteins and their PTMs (Ryan et al., 2010; Howery et al., 2012). Such analysis will make it easier to identify nearly-stoichiometric (and potentially important) PTM events from trace ones under different conditions, not an easy task using bottom-up MS. Native MS is capable of analyzing certain intact membrane protein complexes, although the technology is still developing, and no one approach works for all protein complexes (reviewed in Mehmood et al., 2015). Native MS analysis of PSII has not yet been demonstrated, but the technique could in theory serve as a complementary method to native gels to characterize the distribution of PSII subcomplexes under various conditions, and their components. This might be particularly useful to address the stoichiometry of accessory proteins and cofactors, and could add a new tool to address the long-standing question of chlorophyll loading in PSII.

## Conclusion

The use of MS has been fueled by improvements in sample preparation methods for analysis of membrane proteins, increasing availability of MS instrumentation, and significant advances in instrument sensitivity, speed, and mass accuracy. Techniques from each of the four pillars of proteomics will continue to be employed to study the PSII life cycle. These techniques have addressed a wide range of questions regarding the composition of PSII complexes, the time-dependent dynamic changes of individual subunits and complexes under different environmental conditions, and the tertiary and quaternary structure of PSII complexes.

Modern MS techniques provide a higher level of detail and confidence than previous methods; examples are identification of a protein's phosphorylation site instead of mere detection of a phosphorylated protein, and identification of specific cross-linked residues instead of only suggestive evidence that two particular proteins are cross-linked to each other. For other applications, the use of MS permits entirely new questions to be asked (e.g., what proteins are present on a proteome-wide scale for a purified PSII complex).

The new information has opened up new questions about function. For example, what are the physiological roles of the many new PTMs that have been identified? What purpose does an accessory protein serve by binding at this particular location on a PSII complex? In some cases, the sensitivity of MS is a potential pitfall: identification of a protein in a PSII sample does not necessarily mean it is a stoichiometric component, or that it associates specifically with the complex at all. Thus, information from MS should be a starting point for more targeted genetic and biochemical studies, and MS is one component of an expanding toolbox for PSII life-cycle research. Rapidly developing MS technology promises continued contributions to this field, which has a wide range of fascinating questions about membrane protein complex composition, dynamics, and structure yet to be answered.

## Author contributions

HP, MG conceived of the article. DW collected data from the literature and drafted the manuscript. HP, MG, and DW revised the manuscript.

### Conflict of interest statement

The authors declare that the research was conducted in the absence of any commercial or financial relationships that could be construed as a potential conflict of interest.

## Abbreviations

*A.thaliana*: *Arabidopsis thaliana*
BS^3^: (bis(sulfosuccinimidyl)suberate)
*C. reinhardtii*: *Chlamydomonas reinhardtii*
DSP: dithiobis(succinimidyl propionate)
DTSSP: 3,3′-dithiobis(sulfosuccinimidyl propionate)
EDC: 1-ethyl-3-(3-dimethylaminopropyl)carbodiimide
ESI: electrospray ionization
FAB: fast atom bombardment
FTICR: Fourier transform ion cyclotron resonance
GEE: glycine ethyl ester
LC: liquid chromatography
LHCII: Light-harvesting complex II
LMM: low-molecular-mass
LTQ: linear ion trap quadrupole
MALDI: matrix-assisted laser desorption ionization
MS: mass spectrometry
*N. tabacum*: *Nicotiana tabacum*
NHS: N-hydroxysuccinimide
OCP: orange carotenoid protein
PBS: phycobilisome
PSII: Photosystem II
PC: plastocyanin
PTM: post-translational modification
QqQ: triple-quadrupole
SCP: small CAB-like protein
*Synechococcus* 7002: *Synechococcus* sp. PCC 7002
*Synechocystis* 6803: *Synechocystis* sp. PCC 6803
*T. elongatus*: *Thermosynechococcus elongatus*
TOF: time-of-flight
WOC: water-oxidizing complex.

## References

[B1] Adir N. Zer H. Shochat S. Ohad I. (2003). Photoinhibition - a historical perspective. Photosynth. Res. 76, 343–370. 10.1023/A:1024969518145 16228592

[B2] Ago H. Adachi H. Umena Y. Tashiro T. Kawakami K. Kamiya N.. (2016). Novel features of eukaryotic Photosystem II revealed by its crystal structure analysis from a red alga. J. Biol. Chem. 291, 5676–5687. 10.1074/jbc.M115.711689 26757821 PMC4786707

[B3] Allahverdiyeva Y. Aro E. M. (2012). Photosynthetic responses of plants to excess light: Mechanisms and conditions for photoinhibition, excess energy dissipation, and repair, in Photosynthesis: Plastid Biology, Energy Conversion and Carbon Assimilation. Advances in Photosynthesis and Respiration, eds Eaton-Rye J. J. Tripathy B. C. Sharkey T. D. (Dordrecht: Springer), 275–297.

[B4] Anderson L. B. Maderia M. Ouellette A. J. A. Putnam-Evans C. Higgins L. Krick T.. (2002). Posttranslational modifications in the CP43 subunit of photosystem II. Proc. Natl. Acad. Sci. U.S.A. 99, 14676–14681. 10.1073/pnas.232591599 12417747 PMC137478

[B5] Anderson L. B. Ouellette A. J. A. Eaton-Rye J. Maderia M. MacCoss M. J. Yates J. R.. (2004). Evidence for a post-translational modification, aspartyl aldehyde, in a photosynthetic membrane protein. J. Am. Chem. Soc. 126, 8399–8405. 10.1021/ja0478781 15237995

[B6] Andersson L. Porath J. (1986). Isolation of phosphoproteins by immobilized metal (Fe^3+^) affinity chromatography. Anal. Biochem. 154, 250–254. 10.1016/0003-2697(86)90523-3 3085541

[B7] Aro E. M. McCaffery S. Anderson J. M. (1993). Photoinhibition and D1 protein degradation in peas acclimated to different growth irradiances. Plant Physiol. 103, 835–843. 12231982 10.1104/pp.103.3.835 PMC159054

[B8] Aro E.-M. Suorsa M. Rokka A. Allahverdiyeva Y. Paakkarinen V. Saleem A.. (2005). Dynamics of photosystem II: a proteomic approach to thylakoid protein complexes. J. Exp. Bot. 56, 347–356. 10.1093/jxb/eri041 15569703

[B9] Aryal U. K. Stöckel J. Krovvidi R. K. Gritsenko M. A. Monroe M. E. Moore R. J.. (2011). Dynamic proteomic profiling of a unicellular cyanobacterium *Cyanothece* ATCC51142 across light-dark diurnal cycles. BMC Syst. Biol. 5:194. 10.1186/1752-0509-5-194 22133144 PMC3261843

[B10] Back J. W. de Jong L. Muijsers A. O. de Koster C. G. (2003). Chemical cross-linking and mass spectrometry for protein structural modeling. J. Mol. Biol. 331, 303–313. 10.1016/S0022-2836(03)00721-6 12888339

[B11] Baena-González E. Aro E.-M. (2002). Biogenesis, assembly and turnover of photosystem II units. Phil. Trans. R. Soc. Lond. B. 357, 1451–1459. 10.1098/rstb.2002.1141 12437884 PMC1693054

[B12] Bantscheff M. Lemeer S. Savitski M. M. Kuster B. (2012). Quantitative mass spectrometry in proteomics: critical review update from 2007 to the present. Anal. Bioanal. Chem. 404, 939–965. 10.1007/s00216-012-6203-4 22772140

[B13] Barber J. Andersson B. (1992). Too much of a good thing—light can be bad for photosynthesis. Trends Biochem. Sci. 17, 61–66. 10.1016/0968-0004(92)90503-2 1566330

[B14] Battchikova N. Angeleri M. Aro E.-M. (2015). Proteomic approaches in research of cyanobacterial photosynthesis. Photosynth. Res. 126, 47–70. 10.1007/s11120-014-0050-4 25359503

[B15] Boehm M. Romero E. Reisinger V. Yu J. Komenda J. Eichacker L. A.. (2011). Investigating the early stages of Photosystem II assembly in *Synechocystis* sp. PCC 6803: Isolation of CP47 and CP43 complexes. J. Biol. Chem. 286, 14812–14819. 10.1074/jbc.M110.207944 21339295 PMC3083219

[B16] Boehm M. Yu J. Reisinger V. Beckova M. Eichacker L. A. Schlodder E.. (2012). Subunit composition of CP43-less photosystem II complexes of *Synechocystis* sp. PCC 6803: implications for the assembly and repair of photosystem II. Phil. Trans. R. Soc. B 367, 3444–3454. 10.1098/rstb.2012.0066 23148271 PMC3497071

[B17] Bonardi V. Pesaresi P. Becker T. Schleiff E. Wagner R. Pfannschmidt T.. (2005). Photosystem II core phosphorylation and photosynthetic acclimation require two different protein kinases. Nature 437, 1179–1182. 10.1038/nature04016 16237446

[B18] Bricker T. M. Mummadisetti M. P. Frankel L. K. (2015). Recent advances in the use of mass spectrometry to examine structure/function relationships in photosystem II. J. Photochem. Photobiol. B 152, 227–246. 10.1016/j.jphotobiol.2015.08.031 26390944

[B19] Bricker T. M. Odom W. R. Queirolo C. B. (1988). Close association of the 33-kDa extrinsic protein with the apoprotein of CPa1 in photosystem II. FEBS Lett. 231, 111–117. 10.1016/0014-5793(88)80713-0

[B20] Bricker T. M. Roose J. L. Fagerlund R. D. Frankel L. K. Eaton-Rye J. J. (2012). The extrinsic proteins of Photosystem II. Biochim. Biophys. Acta 1817, 121–142. 10.1016/j.bbabio.2011.07.006 21801710

[B21] Brunner A. M. Lossl P. Liu F. Huguet R. Mullen C. Yamashita M. (2015). Benchmarking multiple fragmentation methods on an Orbitrap Fusion for top-down phospho-proteoform characterization. Anal. Chem. 87, 4152–4158. 10.1021/acs.analchem.5b00162 25803405

[B22] Buncherd H. Roseboom W. de Koning L. J. de Koster C. G. de Jong L. (2014). A gas phase cleavage reaction of cross-linked peptides for protein complex topology studies by peptide fragment fingerprinting from large sequence database. J. Proteomics 108, 65–77. 10.1016/j.jprot.2014.05.003 24840472

[B23] Caffarri S. Kouřil R. Kereiche S. Boekema E. J. Croce R. (2009). Functional architecture of higher plant photosystem II supercomplexes. EMBO J. 28, 3052–3064. 10.1038/emboj.2009.232 19696744 PMC2760109

[B24] Chen X. Wei S. Ji Y. Guo X. Yang F. (2015). Quantitative proteomics using SILAC: principles, applications, and developments. Proteomics 15, 3175–3192. 10.1002/pmic.201500108 26097186

[B25] Chu F. Mahrus S. Craik C. S. Burlingame A. L. (2006). Isotope-coded and affinity-tagged cross-linking (ICATXL): An efficient strategy to probe protein interaction surfaces. J. Am. Chem. Soc. 128, 10362–10363. 10.1021/ja0614159 16895390

[B26] Clarke A. K. Soitamo A. Gustafsson P. Öquist G. (1993). Rapid interchange between two distinct forms of cyanobacterial photosystem II reaction center protein D1 in response to photoinhibition. Proc. Natl. Acad. Sci. U.S.A. 90, 9973–9977. 10.1073/pnas.90.21.9973 8234343 PMC47695

[B27] Clegg C. Hayes D. (1974). Identification of neighboring proteins in ribosomes of *Escherichia coli*- Topographical study with crosslinking reagent dimethyl suberimidate. Eur. J. Biochem. 42, 21–28. 10.1111/j.1432-1033.1974.tb03309.x 4598101

[B28] Cormann K. U. Möller M. Nowaczyk M. M. (2016). Critical assessment of protein cross-linking and molecular docking: an updated model for the interaction between Photosystem II and Psb27. Front. Plant Sci. 7:157. 10.3389/fpls.2016.00157 26925076 PMC4758025

[B29] Dasgupta J. Ananyev G. Dismukes G. C. (2008). Photoassembly of the water-oxidizing complex in photosystem II. Coord. Chem. Rev. 252, 347–360. 10.1016/j.ccr.2007.08.022 19190725 PMC2597823

[B30] Dedner N. Meyer H. E. Ashton C. Wildner G. F. (1988). N-terminal sequence analysis of the 8 kDa protein in *Chlamydomonas reinhardii*: localization of the phosphothreonine. FEBS Lett. 236, 77–82. 10.1016/0014-5793(88)80288-6

[B31] Dobáková M. Sobotka R. Tichý M. Komenda J. (2009). Psb28 protein is involved in the biogenesis of the Photosystem II inner antenna CP47 (PsbB) in the cyanobacterium *Synechocystis* sp. PCC 6803. Plant Physiol. 149, 1076–1086. 10.1104/pp.108.130039 19036835 PMC2633855

[B32] Dobáková M. Tichý M. Komenda J. (2007). Role of the PsbI protein in photosystem II assembly and repair in the cyanobacterium *Synechocystis* sp. PCC 6803. Plant Physiol. 145, 1681–1691. 10.1104/pp.107.107805 17921338 PMC2151680

[B33] Dreaden T. M. Chen J. Rexroth S. Barry B. A. (2011). N-formylkynurenine as a marker of high light stress in photosynthesis. J. Biol. Chem. 286, 22632–22641. 10.1074/jbc.M110.212928 21527632 PMC3121407

[B34] Enami I. Kamo M. Ohta H. Takahashi S. Miura T. Kusayanagi M. (1998). Intramolecular cross-linking of the extrinsic 33-kDa protein leads to loss of oxygen evolution but not its ability of binding to Photosystem II and stabilization of the manganese cluster. J. Biol. Chem. 273, 4629–4634. 10.1074/jbc.273.8.4629 9468521

[B35] Enami I. Satoh K. Katoh S. (1987). Crosslinking between the 33 kDa extrinsic protein and the 47 kDa chlorophyll-carrying protein of the PSII reaction center core complex. FEBS Lett. 226, 161–165. 10.1016/0014-5793(87)80571-9

[B36] Fagerlund R. D. Eaton-Rye J. J. (2011). The lipoproteins of cyanobacterial photosystem II. J. Photochem. Photobiol. B 104, 191–203. 10.1016/j.jphotobiol.2011.01.022 21349737

[B37] Ficarro S. B. McCleland M. L. Stukenberg P. T. Burke D. J. Ross M. M. Shabanowitz J.. (2002). Phosphoproteome analysis by mass spectrometry and its application to *Saccharomyces cerevisiae*. Nat. Biotechnol. 20, 301–305. 10.1038/nbt0302-301 11875433

[B38] Frankel L. K. Bricker T. M. (1992). Interaction of CPa-1 with the manganese-stabilizing protein of Photosystem II: identification of domains on CPa-1 which are shielded from N-hydroxysuccinimide biotinylation by the manganese-stabilizing protein. Biochemistry 31, 11059–11064. 10.1021/bi00160a015 1445844

[B39] Frankel L. K. Bricker T. M. (1995). Interaction of the 33-kDa extrinsic protein with Photosystem II: identification of domains on the 33-kDa protein that are shielded from NHS-biotinylation by Photosystem II. Biochemistry 34, 7492–7497. 10.1021/bi00022a024 7779793

[B40] Frankel L. K. Cruz J. A. Bricker T. M. (1999). Carboxylate groups on the manganese-stabilizing protein are required for its efficient binding to Photosystem II. Biochemistry 38, 14271–14278. 10.1021/bi991366v 10572001

[B41] Frankel L. K. Sallans L. Bellamy H. Goettert J. S. Limbach P. A. Bricker T. M. (2013a). Radiolytic mapping of solvent-contact surfaces in Photosystem II of higher plants: experimental identification of putative water channels within the photosystem. J. Biol. Chem. 288, 23565–23572. 10.1074/jbc.M113.487033 23814046 PMC3949330

[B42] Frankel L. K. Sallans L. Limbach P. A. Bricker T. M. (2013b). Oxidized amino acid residues in the vicinity of Q_*A*_ and Pheo_*D*1_ of the Photosystem II reaction center: putative generation sites of reducing-side reactive oxygen species. PLoS ONE 8:e58042 10.1371/journal.pone.0058042 23469138 PMC3585169

[B43] Frankel L. K. Sallans L. Limbach P. Bricker T. M. (2012). Identification of oxidized amino acid residues in the vicinity of the Mn_4_CaO_5_ cluster of Photosystem II: implications for the identification of oxygen channels within the photosystem. Biochemistry 51, 6371–6377. 10.1021/bi300650n 22827410 PMC3448023

[B44] Frese C. K. Altelaar A. F. M. van den Toorn H. Nolting D. Griep-Raming J. Heck A. J. R.. (2012). Toward full peptide sequence coverage by dual fragmentation combining electron-transfer and higher-energy collision dissociation tandem mass spectrometry. Anal. Chem. 84, 9668–9673. 10.1021/ac3025366 23106539

[B45] Fristedt R. Granath P. Vener A. V. (2010). A protein phosphorylation threshold for functional stacking of plant photosynthetic membranes. PLoS ONE 5:e10963. 10.1371/journal.pone.0010963 20532038 PMC2881038

[B46] Fristedt R. Vener A. V. (2011). High light induced disassembly of Photosystem II supercomplexes in *Arabidopsis* requires STN7-dependent phosphorylation of CP29. PLoS ONE 6:e24565. 10.1371/journal.pone.0024565 21915352 PMC3168523

[B47] Fristedt R. Willig A. Granath P. Crevecoeur M. Rochaix J.-D. Vener A. V. (2009). Phosphorylation of Photosystem II controls functional macroscopic folding of photosynthetic membranes in *Arabidopsis*. Plant Cell 21, 3950–3964. 10.1105/tpc.109.069435 20028840 PMC2814517

[B48] Fritzsche R. Ihling C. H. Götze M. Sinz A. (2012). Optimizing the enrichment of cross-linked products for mass spectrometric protein analysis. Rapid Commun. Mass Spectrom. 26, 653–658. 10.1002/rcm.6150 22328219

[B49] Gabdulkhakov A. Guskov A. Broser M. Kern J. Mueh F. Saenger W. (2009). Probing the accessibility of the Mn_4_Ca cluster in Photosystem II: channels calculation, noble gas derivatization, and cocrystallization with DMSO. Structure 17, 1223–1234. 10.1016/j.str.2009.07.010 19748343

[B50] Galetskiy D. Lohscheider J. N. Kononikhin A. S. Popov I. A. Nikolaev E. N. Adamska I. (2011a). Mass spectrometric characterization of photooxidative protein modifications in *Arabidopsis thaliana* thylakoid membranes. Rapid Commun. Mass Spectrom. 25, 184–190. 10.1002/rcm.4855 21154902

[B51] Galetskiy D. Lohscheider J. N. Kononikhin A. S. Popov I. A. Nikolaev E. N. Adamska I. (2011b). Phosphorylation and nitration levels of photosynthetic proteins are conversely regulated by light stress. Plant Mol. Biol. 77, 461–473. 10.1007/s11103-011-9824-7 21901528

[B52] Gao S. Gu W. Xiong Q. Ge F. Xie X. Li J.. (2015). Desiccation enhances phosphorylation of PSII and affects the distribution of protein complexes in the thylakoid membrane. Physiol. Plant. 153, 492–502. 10.1111/ppl.12258 25132456

[B53] Gau B. Garai K. Frieden C. Gross M. L. (2011). Mass spectrometry-based protein footprinting characterizes the structures of oligomeric apolipoprotein E2, E3, and E4. Biochemistry 50, 8117–8126. 10.1021/bi200911c 21848287 PMC3177987

[B54] Geiger T. Cox J. Mann M. (2010). Proteomics on an Orbitrap benchtop mass spectrometer using all-ion fragmentation. Mol. Cell. Proteomics 9, 2252–2261. 10.1074/mcp.M110.001537 20610777 PMC2953918

[B55] Golden S. S. Brusslan J. Haselkorn R. (1986). Expression of a family of *psbA* genes encoding a photosystem II polypeptide in the cyanobacterium *Anacystis nidulans* R2. EMBO J. 5, 2789–2798. 3098559 10.1002/j.1460-2075.1986.tb04569.x PMC1167224

[B56] Gómez S. M. Bil' K. Y. Aguilera R. Nishio J. N. Faull K. F. Whitelegge J. P. (2003). Transit peptide cleavage sites of integral thylakoid membrane proteins. Mol. Cell. Proteomics 2, 1068–1085. 10.1074/mcp.M300062-MCP200 12902551

[B57] Gómez S. M. Nishio J. N. Faull K. F. Whitelegge J. P. (2002). The chloroplast grana proteome defined by intact mass measurements from liquid chromatography mass spectrometry. Mol. Cell. Proteomics 1, 46–59. 10.1074/mcp.M100007-MCP200 12096140

[B58] Gómez S. M. Park J. J. Zhu J. Whitelegge J. P. Thornber J. P. (1998). Isolation and characterization of a novel xanthophyll-rich pigment-protein complex from spinach, in Photosynthesis: Mechanisms and Effects, Vol. 1,” ed Garab G. (Dordrecht: Kluwer Academic Publishers), 353–356.

[B59] Gouw J. W. Krijgsveld J. Heck A. J. R. (2010). Quantitative proteomics by metabolic labeling of model organisms. Mol. Cell. Proteomics 9, 11–24. 10.1074/mcp.R900001-MCP200 19955089 PMC2808257

[B60] Granvogl B. Zoryan M. Plöscher Eichacker, L. A. (2008). Localization of 13 one-helix integral membrane proteins in Photosystem II subcomplexes. Anal. Biochem. 383, 279–288. 10.1016/j.ab.2008.08.038 18804444

[B61] Grasse N. Mamedov F. Becker K. Styring S. Rögner M. Nowaczyk M. M. (2011). Role of novel dimeric Photosystem II (PSII)-Psb27 protein complex in PSII repair. J. Biol. Chem. 286, 29548–29555. 10.1074/jbc.M111.238394 21737447 PMC3190995

[B62] Grossmann J. Roschitzki B. Panse C. Fortes C. Barkow-Oesterreicher S. Rutishauser D.. (2010). Implementation and evaluation of relative and absolute quantification in shotgun proteomics with label-free methods. J. Proteomics 73, 1740–1746. 10.1016/j.jprot.2010.05.011 20576481

[B63] Götze M. Pettelkau J. Fritzsche R. Ihling C. H. Schaefer M. Sinz A. (2015). Automated assignment of MS/MS cleavable cross-links in protein 3D-structure analysis. J. Am. Soc. Mass. Spectrom. 26, 83–97. 10.1007/s13361-014-1001-1 25261217

[B64] Götze M. Pettelkau J. Schaks S. Bosse K. Ihling C. Krauth F.. (2012). StavroX-A Software for analyzing crosslinked products in protein interaction studies. J. Am. Soc. Mass. Spectrom. 23, 76–87. 10.1007/s13361-011-0261-2 22038510

[B65] Guerreiro A. C. L. Benevento M. Lehmann R. van Breukelen B. Post H. Giansanti P. (2014). Daily rhythms in the cyanobacterium *Synechococcus elongatus* probed by high-resolution mass spectrometry-based proteomics reveals a small defined set of cyclic proteins. Mol. Cell. Proteomics 13, 2042–2055. 10.1074/mcp.M113.035840 24677030 PMC4125736

[B66] Guo J. Nguyen A. Y. Dai Z. Su D. Gaffrey M. J. Moore R. J.. (2014). Proteome-wide light/dark modulation of thiol oxidation in cyanobacteria revealed by quantitative site-specific redox proteomics. Mol. Cell. Proteomics 13, 3270–3285. 10.1074/mcp.M114.041160 25118246 PMC4256482

[B67] Guskov A. Kern J. Gabdulkhakov A. Broser M. Zouni A. Saenger W. (2009). Cyanobacterial photosystem II at 2.9-Å resolution and the role of quinones, lipids, channels and chloride. Nat. Struct. Mol. Biol. 16, 334–342. 10.1038/nsmb.1559 19219048

[B68] Han K. C. Shen J. R. Ikeuchi M. Inoue Y. (1994). Chemical cross-linking studies of extrinsic proteins in cyanobacterial photosystem II. FEBS Lett. 355, 121–124. 10.1016/0014-5793(94)01182-6 7982483

[B69] Haniewicz P. De Sanctis D. Büchel C. Schröder W. P. Loi M. C. Kieselbach T.. (2013). Isolation of monomeric photosystem II that retains the subunit PsbS. Photosynth. Res. 118, 199–207. 10.1007/s11120-013-9914-2 23975205 PMC3825537

[B70] Haniewicz P. Floris D. Farci D. Kirkpatrick J. Loi M. C. Büchel C. (2015). Isolation of plant Photosystem II complexes by fractional solubilization. Front. Plant Sci. 6:1100. 10.3389/fols.2015.01100 26697050 PMC4674563

[B71] Hansson M. Dupuis T. Stromquist R. Andersson B. Vener A. V. Carlberg I. (2007). The mobile thylakoid phosphoprotein TSP9 interacts with the light-harvesting complex II and the peripheries of both photosystems. J. Biol. Chem. 282, 16214–16222. 10.1074/jbc.M605833200 17400553

[B72] Harrer R. Bassi R. Testi M. G. Schäfer C. (1998). Nearest-neighbor analysis of a photosystem II complex from *Marchantia polymorpha* L. (liverwort), which contains reaction center and antenna proteins. Eur. J. Biochem. 255, 196–205. 10.1046/j.1432-1327.1998.2550196.x 9692919

[B73] Heinemeyer J. Eubel H. Wehmhoner D. Jansch L. Braun H. P. (2004). Proteomic approach to characterize the supramolecular organization of photosystems in higher plants. Phytochemistry 65, 1683–1692. 10.1016/j.phytochem.2004.04.022 15276430

[B74] Heinz S. Liauw P. Nickelsen J. Nowaczyk M. (2016). Analysis of photosystem II biogenesis in cyanobacteria. Biochim. Biophys. Acta 1857, 274–287. 10.1016/j.bbabio.2015.11.007 26592144

[B75] Herbstová M. Tietz S. Kinzel C. Turkina M. Kirchhoff H. (2012). Architectural switch in plant photosynthetic membranes induced by light stress. Proc. Natl. Acad. Sci. U.S.A. 109, 20130–20135. 10.1073/pnas.1214265109 23169624 PMC3523818

[B76] Ho F. M. Styring S. (2008). Access channels and methanol binding site to the CaMn_4_ cluster in Photosystem II based on solvent accessibility simulations, with implications for substrate water access. Biochim. Biophys. Acta 1777, 140–153. 10.1016/j.bbabio.2007.08.009 17964532

[B77] Hoare D. G. Koshland D. E. (1967). A method for quantitative modification and estimation of carboxylic acid groups in proteins. J. Biol. Chem. 242, 2447–2453. 6026234

[B78] Hoopmann M. R. Zelter A. Johnson R. S. Riffle M. MacCoss M. J. Davis T. N.. (2015). Kojak: efficient analysis of chemically cross-linked protein complexes. J. Proteome Res. 14, 2190–2198. 10.1021/pr501321h 25812159 PMC4428575

[B79] Howery A. E. Elvington S. Abraham S. J. Choi K. H. Dworschak-Simpson S. Phillips S.. (2012). A designed inhibitor of a CLC antiporter blocks function through a unique binding mode. Chem. Biol. 19, 1460–1470. 10.1016/j.chembiol.2012.09.017 23177200 PMC3508466

[B80] Huber C. G. Walcher W. Timperio A. M. Troiani S. Porceddu A. Zolla L. (2004). Multidimensional proteomic analysis of photosynthetic membrane proteins by liquid extraction-ultracentrifugation-liquid chromatography-mass spectrometry. Proteomics 4, 3909–3920. 10.1002/pmic.200400823 15449339

[B81] Ido K. Kakiuchi S. Uno C. Nishimura T. Fukao Y. Noguchi T.. (2012). The conserved His-144 in the PsbP protein is important for the interaction between the PsbP N-terminus and the Cyt *b*_559_ subunit of Photosystem II. J. Biol. Chem. 287, 26377–26387. 10.1074/jbc.M112.385286 22707728 PMC3406721

[B82] Ido K. Nield J. Fukao Y. Nishimura T. Sato F. Ifuku K. (2014). Cross-linking evidence for multiple interactions of the PsbP and PsbQ proteins in a higher plant Photosystem II supercomplex. J. Biol. Chem. 289, 20150–20157. 10.1074/jbc.M114.574822 24914208 PMC4106330

[B83] Ikeuchi M. Inoue Y. (1988). A new 4.8-kDa polypeptide instrinsic to the PS II reaction center, as revealed by modified SDS-PAGE with improved resolution of low-molecular-weight proteins. Plant Cell Physiol. 29, 1233–1239.

[B84] Inagaki N. Yamamoto Y. Satoh K. (2001). A sequential two-step proteolytic process in the carboxyl-terminal truncation of precursor D1 protein in *Synechocystis* sp. PCC6803. FEBS Lett. 509, 197–201. 10.1016/S0014-5793(01)03180-5 11741588

[B85] Ingle R. A. Schmidt U. G. Farrant J. M. Thomson J. A. Mundree S. G. (2007). Proteomic analysis of leaf proteins during dehydration of the resurrection plant *Xerophyta viscosa*. Plant Cell Environ. 30, 435–446. 10.1111/j.1365-3040.2006.01631.x 17324230

[B86] Inoue-Kashino N. Kashino Y. Orii H. Satoh K. Terashima I. Pakrasi H. B. (2011). S4 protein Sll1252 is necessary for energy balancing in photosynthetic electron transport in *Synechocystis* sp. PCC 6803. Biochemistry 50, 329–339. 10.1021/bi101077e 21141807

[B87] Irrgang K. D. Shi L. X. Funk C. Schröder W. P. (1995). A nuclear-encoded subunit of the Photosystem II reaction center. J. Biol. Chem. 270, 17588–17593. 7615565 10.1074/jbc.270.29.17588

[B88] Iwai M. Suzuki T. Dohmae N. Inoue Y. Ikeuchi M. (2007). Absence of the PsbZ subunit prevents association of PsbK and Ycf12 with the PSII complex in the thermophilic cyanobacterium *Thermosynechococcus elongatus* BP-1. Plant Cell Physiol. 48, 1758–1763. 10.1093/pcp/pcm148 17967798

[B89] Järvi S. Suorsa M. Aro E.-M. (2015). Photosystem II repair in plant chloroplasts - Regulation, assisting proteins and shared components with photosystem II biogenesis. Biochim. Biophys. Acta 1847, 900–909. 10.1016/j.bbabio.2015.01.006 25615587

[B90] Järvi S. Suorsa M. Paakkarinen V. Aro E.-M. (2011). Optimized native gel systems for separation of thylakoid protein complexes: novel super- and mega-complexes. Biochem J. 439, 207–214. 10.1042/BJ20102155 21707535

[B91] Kang S. Mou L. Lanman J. Velu S. Brouillette W. Prevelige P. E.. (2009). Synthesis of biotin-tagged chemical cross-linkers and their applications for mass spectrometry. Rapid Commun. Mass Spectrom. 23, 1719–1726. 10.1002/rcm.4066 19412923 PMC2748246

[B92] Kao A. Chiu C.-l. Vellucci D. Yang Y. Patel V. R. Guan S. (2011). Development of a novel cross-linking strategy for fast and accurate identification of cross-linked peptides of protein complexes. Mol. Cell. Proteomics 10:002212 10.1074/mcp.M110.002212 20736410 PMC3013449

[B93] Kashino Y. Lauber W. M. Carroll J. A. Wang Q. J. Whitmarsh J. Satoh K. (2002). Proteomic analysis of a highly active photosystem II preparation from the cyanobacterium *Synechocystis* sp. PCC 6803 reveals the presence of novel polypeptides. Biochemistry 41, 8004–8012. 10.1021/bi026012+ 12069591

[B94] Kasson T. M. D. Rexroth S. Barry B. A. (2012). Light-induced oxidative stress, N-formylkynurenine, and oxygenic photosynthesis. PLoS ONE 7:42220. 10.1371/journal.pone.0042220 22860088 PMC3409137

[B95] Kereïche S. Kouřil R. Oostergetel G. T. Fusetti F. Boekema E. J. Doust A. B. (2008). Association of chlorophyll a/c_2_ complexes to photosystem I and photosystem II in the cryptophyte *Rhodomonas* CS24. Biochim. Biophys. Acta 1777, 1122–1128. 10.1016/j.bbabio.2008.04.045 18513489

[B96] Knoppová J. Sobotka R. Tichý M. Yu J. Konik P. Halada P.. (2014). Discovery of a chlorophyll binding protein complex involved in the early steps of Photosystem II assembly in *Synechocystis*. Plant Cell 26, 1200–1212. 10.1105/tpc.114.123919 24681620 PMC4001378

[B97] Komenda J. Hassan H. A. G. Diner B. A. Debus R. J. Barber J. Nixon P. J. (2000). Degradation of the Photosystem II D1 and D2 proteins in different strains of the cyanobacterium *Synechocystis* PCC 6803 varying with respect to the type and level of *psbA* transcript. Plant Mol. Biol. 42, 635–645. 10.1023/A:1006305308196 10809009

[B98] Komenda J. Knoppová J. Kopečná J. Sobotka R. Halada P. Yu J.. (2012a). The Psb27 assembly factor binds to the CP43 complex of Photosystem II in the cyanobacterium *Synechocystis* sp. PCC 6803. Plant Physiol. 158, 476–486. 10.1104/pp.111.184184 22086423 PMC3252115

[B99] Komenda J. Kuviková S. Granvogl B. Eichacker L. A. Diner B. A. Nixon P. J. (2007). Cleavage after residue Ala352 in the C-terminal extension is an early step in the maturation of the D1 subunit of Photosystem II in *Synechocystis* PCC 6803. Biochim. Biophys. Acta 1767, 829–837. 10.1016/j.bbabio.2007.01.005 17300742

[B100] Komenda J. Nickelsen J. Tichý M. Prášil O. Eichacker L. Nixon P. J. (2008). The cyanobacterial homologue of HCF136/YCF48 is a component of an early Photosystem II assembly complex and is important for both the efficient assembly and repair of Photosystem II in Synechocystis sp. PCC 6803. J. Biol. Chem. 283, 22390–22399. 10.1074/jbc.M801917200 18550538

[B101] Komenda J. Reisinger V. Müller B. C. Dobáková M. Granvogl B. Eichacker L. A. (2004). Accumulation of the D2 protein is a key regulatory step for assembly of the Photosystem II reaction center complex in *Synechocystis* PCC 6803. J. Biol. Chem. 279, 48620–48629. 10.1074/jbc.M405725200 15347679

[B102] Komenda J. Sobotka R. Nixon P. J. (2012b). Assembling and maintaining the Photosystem II complex in chloroplasts and cyanobacteria. Curr. Opin. Plant Biol. 15, 245–251. 10.1016/j.pbi.2012.01.017 22386092

[B103] Komenda J. Tichý M. Eichacker L. A. (2005). The PsbH protein is associated with the inner antenna CP47 and facilitates D1 processing and incorporation into PSII in the cyanobacterium *Synechocystis* PCC 6803. Plant Cell Physiol. 46, 1477–1483. 10.1093/pcp/pci159 15970599

[B104] Kós P. B. Deák Z. Cheregi O. Vass I. (2008). Differential regulation of *psbA* and *psbD* gene expression, and the role of the different D1 protein copies in the cyanobacterium *Thermosynechococcus elongatus* BP-1. Biochim. Biophys. Acta 1777, 74–83. 10.1016/j.bbabio.2007.10.015 18053792

[B105] Kouřil R. Dekker J. P. Boekema E. J. (2012). Supramolecular organization of photosystem II in green plants. Biochim. Biophys. Acta 1817, 2–12. 10.1016/j.bbabio.2011.05.024 21723248

[B106] Kufryk G. Hernandez-Prieto M. A. Kieselbach T. Miranda H. Vermaas W. Funk C. (2008). Association of small CAB-like proteins (SCPs) of *Synechocystis* sp. PCC 6803 with Photosystem II. Photosynth. Res. 95, 135–145. 10.1007/s11120-007-9244-3 17912610

[B107] Laganowsky A. Gómez S. M. Whitelegge J. P. Nishio J. N. (2009). Hydroponics on a chip: analysis of the Fe deficient *Arabidopsis* thylakoid membrane proteome. J. Proteomics 72, 397–415. 10.1016/j.jprot.2009.01.024 19367733

[B108] Leitner A. Reischl R. Walzthoeni T. Herzog F. Bohn S. Förster F.. (2012). Expanding the chemical cross-linking toolbox by the use of multiple proteases and enrichment by size exclusion chromatography. Mol. Cell. Proteomics 11:M111.014126. 10.1074/mcp.M111.014126 22286754 PMC3316732

[B109] Lemeille S. Rochaix J.-D. (2010). State transitions at the crossroad of thylakoid signalling pathways. Photosynth. Res. 106, 33–46. 10.1007/s11120-010-9538-8 20217232

[B110] Lemeille S. Turkina M. Vener A. V. Rochaix J.-D. (2010). Stt7-dependent phosphorylation during state transitions in the green alga *Chlamydomonas reinhardtii*. Mol. Cell. Proteomics 9, 1281–1295. 10.1074/mcp.M000020-MCP201 20124224 PMC2877987

[B111] Li X.-J. Yang M.-F. Zhu Y. Liang Y. Shen S.-H. (2011). Proteomic analysis of salt stress responses in rice shoot. J. Plant Biol. 54, 384–395. 10.1007/s12374-011-9173-8

[B112] Liu H. Chen J. Huang R.-C. Weisz D. Gross M. L. Pakrasi H. B. (2013a). Mass spectrometry-based footprinting reveals structural dynamics of Loop E of the chlorophyll-binding protein CP43 during Photosystem II assembly in the cyanobacterium *Synechocystis* 6803. J. Biol. Chem. 288, 14212–14220. 10.1074/jbc.M113.467613 23546881 PMC3656277

[B113] Liu H. Huang R. Y.-C. Chen J. Gross M. L. Pakrasi H. B. (2011a). Psb27, a transiently associated protein, binds to the chlorophyll binding protein CP43 in photosystem II assembly intermediates. Proc. Natl. Acad. Sci. U.S.A. 108, 18536–18541. 10.1073/pnas.1111597108 22031695 PMC3215006

[B114] Liu H. Roose J. L. Cameron J. C. Pakrasi H. B. (2011b). A genetically tagged Psb27 protein allows purification of two consecutive Photosystem II (PSII) assembly intermediates in *Synechocystis* 6803, a cyanobacterium. J. Biol. Chem. 286, 24865–24871. 10.1074/jbc.M111.246231 21592967 PMC3137061

[B115] Liu H. Weisz D. A. Pakrasi H. B. (2015). Multiple copies of the PsbQ protein in a cyanobacterial photosystem II assembly intermediate complex. Photosynth. Res. 126, 375–383. 10.1007/s11120-015-0123-z 25800517

[B116] Liu H. Zhang H. King J. Wolf N. Prado M. Gross M. L.. (2014a). Mass spectrometry footprinting reveals the structural rearrangements of cyanobacterial orange carotenoid protein upon light activation. Biochim. Biophys. Acta 1837, 1955–1963. 10.1016/j.bbabio.2014.09.004 25256653

[B117] Liu H. Zhang H. Niedzwiedzki D. Prado M. He G. Gross M. L.. (2013b). Phycobilisomes supply excitations to both photosystems in a megacomplex in cyanobacteria. Science 342, 1104–1107. 10.1126/science.1242321 24288334 PMC3947847

[B118] Liu H. Zhang H. Orf G. S. Lu Y. Jiang J. King J. D.. (2016). Dramatic domain rearrangements of the cyanobacterial orange carotenoid protein upon photoactivation. Biochemistry 55, 1003–1009. 10.1021/acs.biochem.6b00013 26848988 PMC5201194

[B119] Liu H. Zhang H. Weisz D. A. Vidavsky I. Gross M. L. Pakrasi H. B. (2014b). MS-based cross-linking analysis reveals the location of the PsbQ protein in cyanobacterial photosystem II. Proc. Natl. Acad. Sci. U.S.A. 111, 4638–4643. 10.1073/pnas.1323063111 24550459 PMC3970497

[B120] Lohrig K. Mueller B. Davydova J. Leister D. Wolters D. A. (2009). Phosphorylation site mapping of soluble proteins: bioinformatical filtering reveals potential plastidic phosphoproteins in *Arabidopsis thaliana*. Planta 229, 1123–1134. 10.1007/s00425-009-0901-y 19238429

[B121] Loll B. Kern J. Saenger W. Zouni A. Biesiadka J. (2005). Towards complete cofactor arrangement in the 3.0 Å resolution structure of photosystem II. Nature 438, 1040–1044. 10.1038/nature04224 16355230

[B122] Lorković Z. J. Schröder W. P. Pakrasi H. B. Irrgang K. D. Herrmann R. G. Oelmüller R. (1995). Molecular characterization of PsbW, a nuclear-encoded component of the Photosystem II reaction center complex in spinach. Proc. Natl. Acad. Sci. U.S.A. 92, 8930–8934. 10.1073/pnas.92.19.8930 7568046 PMC41081

[B123] Lundgren D. H. Hwang S.-I. Wu L. Han D. K. (2010). Role of spectral counting in quantitative proteomics. Expert Rev. Proteomics 7, 39–53. 10.1586/EPR.09.69 20121475

[B124] Mabbitt P. D. Wilbanks S. M. Eaton-Rye J. J. (2014). Structure and function of the hydrophilic Photosystem II assembly proteins: Psb27, Psb28 and Ycf48. Plant Physiol. Bioch. 81, 96–107. 10.1016/j.plaphy.2014.02.013 24656878

[B125] Mädler S. Bich C. Touboul D. Zenobi R. (2009). Chemical cross-linking with NHS esters: a systematic study on amino acid reactivities. J. Mass Spectrom. 44, 694–706. 10.1002/jms.1544 19132714

[B126] Meades G. D. McLachlan A. Sallans L. Limbach P. A. Frankel L. K. Bricker T. M. (2005). Association of the 17-kDa extrinsic protein with Photosystem II in higher plants. Biochemistry 44, 15216–15221. 10.1021/bi051704u 16285724

[B127] Mehmood S. Allison T. M. Robinson C. V. (2015). Mass spectrometry of protein complexes: from origins to applications. Annu. Rev. Phys. Chem. 66, 453–474. 10.1146/annurev-physchem-040214-121732 25594852

[B128] Michel H. Hunt D. F. Shabanowitz J. Bennett J. (1988). Tandem mass spectrometry reveals that three Photosystem II proteins of spinach chloroplasts contain *N*-acetyl-*O*-phosphothreonine at their NH_2_ termini. J. Biol. Chem. 263, 1123–1130. 3121625

[B129] Michel H. P. Bennett J. (1987). Identification of the phosphorylation site of an 8.3 kDa protein from photosystem II of spinach. FEBS Lett. 212, 103–108. 10.1016/0014-5793(87)81565-X

[B130] Minagawa J. (2011). State transitions-The molecular remodeling of photosynthetic supercomplexes that controls energy flow in the chloroplast. Biochim. Biophys. Acta 1807, 897–905. 10.1016/j.bbabio.2010.11.005 21108925

[B131] Miura T. Shen J. R. Takahashi S. Kamo M. Nakamura E. Ohta H.. (1997). Identification of domains on the extrinsic 33-kDa protein possibly involved in electrostatic interaction with Photosystem II complex by means of chemical modification. J. Biol. Chem. 272, 3788–3798. 9013637 10.1074/jbc.272.6.3788

[B132] Müller B. Eichacker L. A. (1999). Assembly of the D1 precursor in monomeric Photosystem II reaction center precomplexes precedes Chlorophyll *a*-triggered accumulation of Reaction Center II in barley etioplasts. Plant Cell 11, 2365–2377. 10.1105/tpc.11.12.2365 10590164 PMC144137

[B133] Müller D. R. Schindler P. Towbin H. Wirth U. Voshol H. Hoving S.. (2001). Isotope tagged cross linking reagents. A new tool in mass spectrometric protein interaction analysis. Anal. Chem. 73, 1927–1934. 10.1021/ac001379a 11354472

[B134] Mullet J. E. Christopher D. A. (1994). Separate photosensory pathways coregulate blue-light/ultraviolet-A-activated *psb*D*-psb*C transcription and light-induced D2 and CP43 degradation in barley (*Hordeum vulgare*) chloroplasts. Plant Physiol. 104, 1119–1129. 10.1104/pp.104.4.1119 8016258 PMC159272

[B135] Mullineaux C. W. (2008). Phycobilisome-reaction centre interaction in cyanobacteria. Photosynth. Res. 95, 175–182. 10.1007/s11120-007-9249-y 17922214

[B136] Mulo P. Sakurai I. Aro E.-M. (2012). Strategies for *psbA* gene expression in cyanobacteria, green algae and higher plants: from transcription to PSII repair. Biochim. Biophys. Acta 1817, 247–257. 10.1016/j.bbabio.2011.04.011 21565160

[B137] Mulo P. Sicora C. Aro E.-M. (2009). Cyanobacterial *psbA* gene family: optimization of oxygenic photosynthesis. Cell. Mol. Life Sci. 66, 3697–3710. 10.1007/s00018-009-0103-6 19644734 PMC2776144

[B138] Mummadisetti M. Frankel L. K. Bellamy H. D. Sallans L. Goettert J. S. Brylinski M.. (2014). Use of protein cross-linking and radiolytic footprinting to elucidate PsbP and PsbQ interactions within higher plant Photosystem II. Proc. Natl. Acad. Sci. U.S.A. 111, 16178–16183. 10.1073/pnas.1415165111 25349426 PMC4234589

[B139] Murray J. W. Barber J. (2007). Structural characteristics of channels and pathways in photosystem II including the identification of an oxygen channel. J. Struct. Biol. 159, 228–237. 10.1016/j.jsb.2007.01.f016 17369049

[B140] Nagao R. Suzuki T. Okumura A. Niikura A. Iwai M. Dohmae N. (2010). Topological analysis of the extrinsic PsbO, PsbP and PsbQ proteins in a green algal PSII complex by cross-linking with a water-soluble carbodiimide. Plant Cell Physiol. 51, 718–727. 10.1093/pcp/pcq042 20375107

[B141] Nahnsen S. Bielow C. Reinert K. Kohlbacher O. (2013). Tools for label-free peptide quantification. Mol. Cell. Proteomics 12, 549–556. 10.1074/mcp.R112.025163 23250051 PMC3591650

[B142] Nakamori H. Yatabe T. Yoon K.-S. Ogo S. (2014). Purification and characterization of an oxygen-evolving photosystem II from *Leptolyngbya* sp. strain O-77. J. Biosci. Bioeng. 118, 119–124. 10.1016/j.jbiosc.2014.01.009 24560665

[B143] Nanba O. Satoh K. (1987). Isolation of a photosystem II reaction center consisting of D-1 and D-2 polypeptides and cytochrome *b*-559. Proc. Natl. Acad. Sci. U.S.A. 84, 109–112. 10.1073/pnas.84.1.109 16593792 PMC304151

[B144] Naumann B. Busch A. Allmer J. Ostendorf E. Zeller M. Kirchhoff H.. (2007). Comparative quantitative proteomics to investigate the remodeling of bioenergetic pathways under iron deficiency in *Chlamydomonas reinhardtii*. Proteomics 7, 3964–3979. 10.1002/pmic.200700407 17922516

[B145] Nickelsen J. Rengstl B. (2013). Photosystem II assembly: from cyanobacteria to plants. Annu. Rev. Plant Biol. 64, 609–635. 10.1146/annurev-arplant-050312-120124 23451783

[B146] Nickelsen J. Rengstl B. Stengel A. Schottkowski M. Soll J. Ankele E. (2011). Biogenesis of the cyanobacterial thylakoid membrane system - an update. FEMS Microbiol. Lett. 315, 1–5. 10.1111/j.1574-6968.2010.02096.x 20831593

[B147] Nixon P. J. Michoux F. Yu J. Boehm M. Komenda J. (2010). Recent advances in understanding the assembly and repair of photosystem II. Ann. Bot. 106, 1–16. 10.3389/fols.2075.07700 20338950 PMC2889791

[B148] Nixon P. J. Trost J. T. Diner B. A. (1992). Role of the carboxy terminus of polypeptide D1 in the assembly of a functional water-oxidizing manganese cluster in Photosystem II of the cyanobacterium *Synechocystis* sp. PCC 6803: assembly requires a free carboxyl group at C-terminal position 344. Biochemistry 31, 10859–10871. 10.1021/bi00159a029 1420199

[B149] Nowaczyk M. M. Hebeler R. Schlodder E. Meyer H. E. Warscheid B. Rögner M. (2006). Psb27, a cyanobacterial lipoprotein, is involved in the repair cycle of Photosystem II. Plant Cell 18, 3121–3131. 10.1105/tpc.106.042671 17114356 PMC1693947

[B150] Nowaczyk M. M. Krause K. Mieseler M. Sczibilanski A. Ikeuchi M. Rögner M. (2012). Deletion of *psbJ* leads to accumulation of Psb27-Psb28 photosystem II complexes in *Thermosynechococcus elongatus*. Biochim. Biophys. Acta 1817, 1339–1345. 10.1016/j.bbabio.2012.02.017 22387395

[B151] Odom W. R. Bricker T. M. (1992). Interaction of CPa-1 with the manganese-stabilizing protein of Photosystem II: identification of domains cross-linked by 1-ethyl-3-[3-(dimethylamino)propyl]carbodiimide. Biochemistry 31, 5616–5620. 10.1021/bi00139a027 1610808

[B152] Ohnishi N. Murata N. (2006). Glycinebetaine counteracts the inhibitory effects of salt stress on the degradation and synthesis of D1 protein during photoinhibition in *Synechococcus* sp. PCC 7942. Plant Physiol. 141, 758–765. 10.1104/pp.106.076976 16632587 PMC1475447

[B153] Ohta H. Suzuki T. Ueno M. Okumura A. Yoshihara S. Shen J. R. (2003). Extrinsic proteins of photosystem II - An intermediate member of the PsbQ protein family in red algal PSII. Eur. J. Biochem. 270, 4156–4163. 10.1046/j.1432-1033.2003.03810.x 14519128

[B154] Owens G. C. Ohad I. (1982). Phosphorylation of *Chlamydomonas reinhardi* chloroplast membrane proteins *in vivo* and *in vitro*. J. Cell. Biol. 93, 712–718. 10.1083/jcb.93.3.712 6811597 PMC2112165

[B155] Owens G. C. Ohad I. (1983). Changes in thylakoid polypeptide phosphorylation during membrane biogenesis in *Chlamydomonas reinhardii* y-1. Biochim. Biophys. Acta 722, 234–241. 10.1016/0005-2728(83)90179-2

[B156] Pagliano C. Chimirri F. Saracco G. Marsano F. Barber J. (2011). One-step isolation and biochemical characterization of a highly active plant PSII monomeric core. Photosynth. Res. 108, 33–46. 10.1007/s11120-011-9650-4 21487931

[B157] Pagliano C. Nield J. Marsano F. Pape T. Barera S. Saracco G.. (2014). Proteomic characterization and three-dimensional electron microscopy study of PSII-LHCII supercomplexes from higher plants. Biochim. Biophys. Acta 1837, 1454–1462. 10.1016/j.bbabio.2013.11.004 24246636

[B158] Paramelle D. Miralles G. Subra G. Martinez J. (2013). Chemical cross-linkers for protein structure studies by mass spectrometry. Proteomics 13, 438–456. 10.1002/pmic.201200305 23255214

[B159] Pearson K. M. Pannell L. K. Fales H. M. (2002). Intramolecular cross-linking experiments on cytochrome c and ribonuclease A using an isotope multiplet method. Rapid Commun. Mass Spectrom. 16, 149–159. 10.1002/rcm.554 11803535

[B160] Pesaresi P. Pribil M. Wunder T. Leister D. (2011). Dynamics of reversible protein phosphorylation in thylakoids of flowering plants: the roles of STN7, STN8 and TAP38. Biochim. Biophys. Acta 1807, 887–896. 10.1016/j.bbabio.2010.08.002 20728426

[B161] Petrotchenko E. V. Borchers C. H. (2010). ICC-CLASS: isotopically-coded cleavable crosslinking analysis software suite. BMC Bioinformatics 11:64. 10.1186/1471-2105-11-64 20109223 PMC2827373

[B162] Petrotchenko E. V. Makepeace K. A. T. Serpa J. J. Borchers C. H. (2014). Analysis of protein structure by cross-linking combined with mass spectrometry. Methods Mol. Biol. 1156, 447–463. 10.1007/978-1-4939-0685-7_30 24792007

[B163] Petrotchenko E. V. Serpa J. J. Borchers C. H. (2011). An isotopically coded CID-cleavable biotinylated cross-linker for structural proteomics. Mol. Cell. Proteomics 10:M110001420. 10.1074/mcp.M110.001420 20622150 PMC3033670

[B164] Plöscher M. Granvogl B. Zoryan M. Reisinger V. Eichacker L. A. (2009). Mass spectrometric characterization of membrane integral low molecular weight proteins from photosystem II in barley etioplasts. Proteomics 9, 625–635. 10.1002/pmic.200800337 19137553

[B165] Plöscher M. Reisinger V. Eichacker L. A. (2011). Proteomic comparison of etioplast and chloroplast protein complexes. J. Proteomics 74, 1256–1265. 10.1016/j.jprot.2011.03.020 21440687

[B166] Pospíšil P. (2009). Production of reactive oxygen species by photosystem II. Biochim. Biophys. Acta 1787, 1151–1160. 10.1016/j.bbabio.2009.05.005 19463778

[B167] Promnares K. Komenda J. Bumba L. Nebesarova J. Vacha F. Tichy M. (2006). Cyanobacterial small chlorophyll-binding protein ScpD (HliB) is located on the periphery of Photosystem II in the vicinity of PsbH and CP47 subunits. J. Biol. Chem. 281, 32705–32713. 10.1074/jbc.M606360200 16923804

[B168] Puthiyaveetil S. Kirchhoff H. (2013). A phosphorylation map of the photosystem II supercomplex C2S2M2. Front. Plant Sci. 4:459 10.3389/fpls.2013.00459 PMC3828554 24298276

[B169] Rabilloud T. Chevallet M. Luche S. Lelong C. (2010). Two-dimensional gel electrophoresis in proteomics: Past, present and future. J. Proteomics 73, 2064–2077. 10.1016/j.jprot.2010.05.016 20685252

[B170] Rappsilber J. (2011). The beginning of a beautiful friendship: cross-linking/mass spectrometry and modelling of proteins and multi-protein complexes. J. Struct. Biol. 173, 530–540. 10.1016/j.jsb.2010.10.014 21029779 PMC3043253

[B171] Rast A. Heinz S. Nickelsen J. (2015). Biogenesis of thylakoid membranes. Biochim. Biophys. Acta 1847, 821–830. 10.1016/j.bbabio.2015.01.007 25615584

[B172] Reiland S. Messerli G. Baerenfaller K. Gerrits B. Endler A. Grossmann J.. (2009). Large-scale Arabidopsis phosphoproteome profiling reveals novel chloroplast kinase substrates and phosphorylation networks. Plant Physiol. 150, 889–903. 10.1104/pp.109.138677 19376835 PMC2689975

[B173] Rengstl B. Knoppová J. Komenda J. Nickelsen J. (2013). Characterization of a *Synechocystis* double mutant lacking the photosystem II assembly factors YCF48 and Sll0933. Planta 237, 471–480. 10.1007/s00425-012-1720-0 22847023

[B174] Rexroth S. Wong C. C. L. Park J. H. Yates J. R. III Barry B. A. (2007). An activated glutamate residue identified in Photosystem II at the interface between the manganese-stabilizing subunit and the D2 polypeptide. J. Biol. Chem. 282, 27802–27809. 10.1074/jbc.M704394200 17666402

[B175] Rinalducci S. Larsen M. R. Mohammed S. Zolla L. (2006). Novel protein phosphorylation site identification in spinach stroma membranes by titanium dioxide microcolumns and tandem mass spectrometry. J. Proteome Res. 5, 973–982. 10.1021/pr050476n 16602705

[B176] Rinner O. Seebacher J. Walzthoeni T. Mueller L. Beck M. Schmidt A.. (2008). Identification of cross-linked peptides from large sequence databases. Nat. Methods 5, 315–318. 10.1038/NMETH.1192 18327264 PMC2719781

[B177] Rintamäki E. Salonen M. Suoranta U. M. Carlberg I. Andersson B. Aro E.-M. (1997). Phosphorylation of Light-harvesting Complex II and Photosystem II core proteins shows different irradiance-dependent regulation *in vivo*: application of phosphothreonine antibodies to analysis of thylakoid phosphoproteins. J. Biol. Chem. 272, 30476–30482. 10.1074/jbc.272.48.30476 9374540

[B178] Rokka A. Suorsa M. Saleem A. Battchikova N. Aro E. M. (2005). Synthesis and assembly of thylakoid protein complexes: multiple assembly steps of photosystem II. Biochem. J. 388, 159–168. 10.1042/BJ20042098 15638811 PMC1186704

[B179] Romanowska E. Wasilewska W. Fristedt R. Vener A. V. Zienkiewicz M. (2012). Phosphorylation of PSII proteins in maize thylakoids in the presence of Pb ions. J. Plant Physiol. 169, 345–352. 10.1016/j.jplph.2011.10.006 22169074

[B180] Roose J. L. Kashino Y. Pakrasi H. B. (2007). The PsbQ protein defines cyanobacterial Photosystem II complexes with highest activity and stability. Proc. Natl. Acad. Sci. U.S.A. 104, 2548–2553. 10.1073/pnas.0609337104 17287351 PMC1892988

[B181] Roose J. L. Pakrasi H. B. (2004). Evidence that D1 processing is required for manganese binding and extrinsic protein assembly into Photosystem II. J. Biol. Chem. 279, 45417–45422. 10.1074/jbc.M408458200 15308630

[B182] Roose J. L. Pakrasi H. B. (2008). The Psb27 protein facilitates manganese cluster assembly in Photosystem II. J. Biol. Chem. 283, 4044–4050. 10.1074/jbc.M708960200 18089572

[B183] Ross P. L. Huang Y. L. N. Marchese J. N. Williamson B. Parker K. Hattan S. (2004). Multiplexed protein quantitation in *Saccharomyces cerevisiae* using amine-reactive isobaric tagging reagents. Mol. Cell. Proteomics 3, 1154–1169. 10.1074/mcp.M400129-MCP200 15385600

[B184] Rowland J. G. Simon W. J. Nishiyama Y. Slabas A. R. (2010). Differential proteomic analysis using iTRAQ reveals changes in thylakoids associated with Photosystem II-acquired thermotolerance in *Synechocystis* sp. PCC 6803. Proteomics 10, 1917–1929. 10.1002/pmic.200900337 20336677

[B185] Ryan C. M. Souda P. Bassilian S. Ujwal R. Zhang J. Abramson J.. (2010). Post-translational modifications of integral membrane proteins resolved by top-down Fourier transform mass spectrometry with collisionally activated dissociation. Mol. Cell. Proteomics 9, 791–803. 10.1074/mcp.M900516-MCP200 20093275 PMC2871414

[B186] Samol I. Shapiguzov A. Ingelsson B. Fucile G. Crevecoeur M. Vener A. V.. (2012). Identification of a Photosystem II phosphatase involved in light acclimation in *Arabidopsis*. Plant Cell 24, 2596–2609. 10.1105/tpc.112.095703 22706287 PMC3406908

[B187] Sander J. Nowaczyk M. Buchta J. Dau H. Vass I. Deák Z.. (2010). Functional characterization and quantification of the alternative PsbA copies in *Thermosynechococcus elongatus* and their role in photoprotection. J. Biol. Chem. 285, 29851–29856. 10.1074/jbc.M110.127142 20663887 PMC2943314

[B188] Schönberg A. Baginsky S. (2012). Signal integration by chloroplast phosphorylation networks: an update. Front. Plant Sci. 3:256 10.3389/fpls.2012.00256 PMC3501822 23181067

[B189] Schottkowski M. Gkalympoudis S. Tzekova N. Stelljes C. Schuenemann D. Ankele E.. (2009). Interaction of the periplasmic PratA factor and the PsbA (D1) protein during biogenesis of Photosystem II in *Synechocystis* sp. PCC 6803. J. Biol. Chem. 284, 1813–1819. 10.1074/jbc.M806116200 19017636

[B190] Seebacher J. Mallick P. Zhang N. Eddes J. Aebersold R. Gelb M. H. (2006). Protein cross-linking analysis using mass spectrometry, isotope-coded cross-linkers, and integrated computational data processing. J. Proteome Res. 5, 2270–2282. 10.1021/pr060154z 16944939

[B191] Seidler A. (1996). Intermolecular and intramolecular interactions of the 33-kDa protein in photosystem II. Eur. J. Biochem. 242, 485–490. 10.1111/j.1432-1033.1996.0485r.x 9022672

[B192] Sharma J. Panico M. Barber J. Morris H. R. (1997a). Characterization of the low molecular weight photosystem II reaction center subunits and their light-induced modifications by mass spectrometry. J. Biol. Chem. 272, 3935–3943. 9020097 10.1074/jbc.272.7.3935

[B193] Sharma J. Panico M. Barber J. Morris H. R. (1997b). Purification and determination of intact molecular mass by electrospray ionization mass spectrometry of the photosystem II reaction center subunits. J. Biol. Chem. 272, 33153–33157. 9407102 10.1074/jbc.272.52.33153

[B194] Sharma J. Panico M. Shipton C. A. Nilsson F. Morris H. R. Barber J. (1997c). Primary structure characterization of the photosystem II D1 and D2 subunits. J. Biol. Chem. 272, 33158–33166. 10.1074/jbc.272.52.33158 9407103

[B195] Shaw J. B. Li W. Holden D. D. Zhang Y. Griep-Raming J. Fellers R. T. (2013). Complete protein characterization using top-down mass spectrometry and ultraviolet photodissociation. J. Am. Chem. Soc. 135, 12646–12651. 10.1021/ja4029654 23697802 PMC3757099

[B196] Shevela D. Messinger J. (2013). Studying the oxidation of water to molecular oxygen in photosynthetic and artificial systems by time-resolved membrane-inlet mass spectrometry. Front. Plant Sci. 4:473. 10.3389/fpls.2013.00473 24324477 PMC3840314

[B197] Shi L.-X. Hall M. Funk C. Schröder W. (2012). Photosystem II, a growing complex: updates on newly discovered components and low molecular mass proteins. Biochim. Biophys. Acta 1817, 13–25. 10.1016/j.bbabio.2011.08.008 21907181

[B198] Shi L.-X. Lorković Z. J. Oelmüller R. Schröder W. P. (2000). The low molecular mass PsbW protein is involved in the stabilization of the dimeric Photosystem II complex in *Arabidopsis thaliana*. J. Biol. Chem. 275, 37945–37950. 10.1074/jbc.M006300200 10950961

[B199] Shi L.-X. Schröder W. P. (1997). Compositional and topological studies of the PsbW protein in spinach thylakoid membrane. Photosynth. Res. 53, 45–53. 10.1023/A:1005830405809

[B200] Silva J. C. Gorenstein M. V. Li G. Z. Vissers J. P. C. Geromanos S. J. (2006). Absolute quantification of proteins by LCMS^E^ - A virtue of parallel MS acquisition. Mol. Cell. Proteomics 5, 144–156. 10.1074/mcp.M500230-MCP200 16219938

[B201] Sinz A. (2014). The advancement of chemical cross-linking and mass spectrometry for structural proteomics: from single proteins to protein interaction networks. Expert Rev. Proteomics 11, 733–743. 10.1586/14789450.2014.960852 25227871

[B202] Stöckel J. Jacobs J. M. Elvitigala T. R. Liberton M. Welsh E. A. Polpitiya A. D.. (2011). Diurnal rhythms result in significant changes in the cellular protein complement in the cyanobacterium *Cyanothece* 51142. PLoS ONE 6:e16680. 10.1371/journal.pone.0016680 21364985 PMC3043056

[B203] Steinback K. E. Bose S. Kyle D. J. (1982). Phosphorylation of the light-harvesting chlorophyll-protein regulates excitation energy distribution between Photosystem II and Photosystem I. Arch. Biochem. Biophys. 216, 356–361. 10.1016/0003-9861(82)90221-1 7049089

[B204] Sugimoto I. Takahashi Y. (2003). Evidence that the PsbK polypeptide is associated with the Photosystem II core antenna complex CP43. J. Biol. Chem. 278, 45004–45010. 10.1074/jbc.M307537200 12939265

[B205] Sugiura M. Boussac A. (2014). Some Photosystem II properties depending on the D1 protein variants in Thermosynechococcus elongatus. Biochim. Biophys. Acta 1837, 1427–1434. 10.1016/j.bbabio.2013.12.011 24388918

[B206] Sugiura M. Iwai E. Hayashi H. Boussac A. (2010a). Differences in the interactions between the subunits of Photosystem II dependent on D1 protein variants in the thermophilic cyanobacterium *Thermosynechococcus elongatus*. J. Biol. Chem. 285, 30008–30018. 10.1074/jbc.M110.136945 20630865 PMC2943282

[B207] Sugiura M. Kato Y. Takahashi R. Suzuki H. Watanabe T. Noguchi T.. (2010b). Energetics in Photosystem II from *Thermosynechococcus elongatus* with a D1 protein encoded by either the psbA_1_ or psbA_3_ gene. Biochim. Biophys. Acta 1797, 1491–1499. 10.1016/j.bbabio.2010.03.022 20362546

[B208] Sugiura M. Koyama K. Umena Y. Kawakami K. Shen J.-R. Kamiya N.. (2013). Evidence for an unprecedented histidine hydroxyl modification on D2-His336 in Photosystem II of *Thermosynechoccocus vulcanus* and *Thermosynechoccocus elongatus*. Biochemistry 52, 9426–9431. 10.1021/bi401213m 24320870

[B209] Sugiyama N. Nakagami H. Mochida K. Daudi A. Tomita M. Shirasu K. (2008). Large-scale phosphorylation mapping reveals the extent of tyrosine phosphorylation in *Arabidopsis*. Mol. Syst. Biol. 4, 193 10.1038/msb.2008.32 18463617 PMC2424297

[B210] Swaisgood H. Natake M. (1973). Effect of carboxyl group modification on some of the enzymatic properties of L-glutamate dehydrogenase. J. Biochem. 74, 77–86. 4354838 10.1093/oxfordjournals.jbchem.a130233

[B211] Takahashi M. Shiraishi T. Asada K. (1988). COOH-terminal residues of D1 and the 44 kDa CPa-2 at spinach photosystem II core complex. FEBS Lett. 240, 6–8. 10.1016/0014-5793(88)80330-2 3056748

[B212] Takamoto K. Chance M. R. (2006). Radiolytic protein footprinting with mass spectrometry to probe the structure of macromolecular complexes. Annu. Rev. Biophys. Biomol. Struct. 35, 251–276. 10.1146/annurev.biophys.35.040405.102050 16689636

[B213] Takasaka K. Iwai M. Umena Y. Kawakami K. Ohmori Y. Ikeuchi M.. (2010). Structural and functional studies on Ycf12 (Psb30) and PsbZ-deletion mutants from a thermophilic cyanobacterium. Biochim. Biophys. Acta 1797, 278–284. 10.1016/j.bbabio.2009.11.001 19917266

[B214] Tal O. Trabelcy B. Gerchman Y. Adir N. (2014). Investigation of phycobilisome subunit interaction interfaces by coupled cross-linking and mass spectrometry. J. Biol. Chem. 289, 33084–33097. 10.1074/jbc.M114.595942 25296757 PMC4246069

[B215] Telfer A. Bishop S. M. Phillips D. Barber J. (1994). Isolated photosynthetic reaction center of Photosystem II as a sensitizer for the formation of singlet oxygen: detection and quantum yield determination using a chemical trapping technique. J. Biol. Chem. 269, 13244–13253. 8175754

[B216] Thangaraj B. Ryan C. M. Souda P. Krause K. Faull K. F. Weber A. P. M.. (2010). Data-directed top-down Fourier-transform mass spectrometry of a large integral membrane protein complex: Photosystem II from *Galdieria sulphuraria*. Proteomics 10, 3644–3656. 10.1002/pmic.201000190 20845333 PMC3517113

[B217] Thelen J. J. Peck S. C. (2007). Quantitative proteomics in plants: choices in abundance. Plant Cell 19, 3339–3346. 10.1105/tpc.107.053991 18055608 PMC2174896

[B218] Thidholm E. Lindström V. Tissier C. Robinson C. Schröder W. P. Funk C. (2002). Novel approach reveals localisation and assembly pathway of the PsbS and PsbW proteins into the photosystem II dimer. FEBS Lett. 513, 217–222. 10.1016/S0014-5793(02)02314-1 11904154

[B219] Thompson A. Schäfer J. Kuhn K. Kienle S. Schwarz J. Schmidt G.. (2003). Tandem mass tags: a novel quantification strategy for comparative analysis of complex protein mixtures by MS/MS. Anal. Chem. 75, 1895–1904. 10.1021/ac0262560 12713048

[B220] Thornton L. E. Ohkawa H. Roose J. L. Kashino Y. Keren N. Pakrasi H. B. (2004). Homologs of plant PsbP and PsbQ proteins are necessary for regulation of Photosystem II activity in the cyanobacterium *Synechopystis* 6803. Plant Cell 16, 2164–2175. 10.1105/tpc.104.023515 15258264 PMC519205

[B221] Tikhonov A. N. (2015). Induction events and short-term regulation of electron transport in chloroplasts: an overview. Photosynth. Res. 125, 65–94. 10.1007/s11120-015-0094-0 25680580

[B222] Tikkanen M. Aro E.-M. (2014). Integrative regulatory network of plant thylakoid energy transduction. Trends Plant Sci. 19, 10–17. 10.1016/j.tplants.2013.09.003 24120261

[B223] Tikkanen M. Grieco M. Kangasjarvi S. Aro E.-M. (2010). Thylakoid protein phosphorylation in higher plant chloroplasts optimizes electron transfer under fluctuating light. Plant Physiol. 152, 723–735. 10.1104/pp.109.150250 19965965 PMC2815896

[B224] Tikkanen M. Nurmi M. Kangasjarvi S. Aro E.-M. (2008a). Core protein phosphorylation facilitates the repair of photodamaged photosystem II at high light. Biochim. Biophys. Acta 1777, 1432–1437. 10.1016/j.bbabio.2008.08.004 18774768

[B225] Tikkanen M. Nurmi M. Suorsa M. Danielsson R. Mamedov F. Styring S.. (2008b). Phosphorylation-dependent regulation of excitation energy distribution between the two photosystems in higher plants. Biochim. Biophys. Acta 1777, 425–432. 10.1016/j.bbabio.2008.02.001 18331820

[B226] Tomo T. Enami I. Satoh K. (1993). Orientation and nearest-neighbor analysis of *psb*I gene product in the photosystem II reaction center complex using bifunctional cross-linkers. FEBS Lett. 323, 15–18. 10.1016/0014-5793(93)81438-6 8495728

[B227] Tsiotis G. Walz T. Spyridaki A. Lustig A. Engel A. Ghanotakis D. (1996). Tubular crystals of a Photosystem II core complex. J. Mol. Biol. 259, 241–248. 10.1006/jmbi.1996.0316 8656426

[B228] Turkina M. V. Kargul J. Blanco-Rivero A. Villarejo A. Barber J. Vener A. V. (2006). Environmentally modulated phosphoproteome of photosynthetic membranes in the green alga *Chlamydomonas reinhardtii*. Mol. Cell Proteomics 5, 1412–1425. 10.1074/mcp.M600066-MCP200 16670252

[B229] Tyystjärvi E. (2013). Photoinhibition of Photosystem II. Int. Rev. Cell. Mol. Biol. 300, 243–303. 10.1016/B978-0-12-405210-9.00007-2 23273864

[B230] Ujihara T. Sakurai I. Mizusawa N. Wada H. (2008). A method for analyzing lipid-modified proteins with mass spectrometry. Anal. Biochem. 374, 429–431. 10.1016/j.ab.2007.11.014 18078799

[B231] Umena Y. Kawakami K. Shen J. Kamiya N. (2011). Crystal structure of oxygen-evolving photosystem II at a resolution of 1.9 Å. Nature 473, 55–60. 10.1038/nature09913 21499260

[B232] Vainonen J. P. Hansson M. Vener A. V. (2005). STN8 protein kinase in *Arabidopsis thaliana* is specific in phosphorylation of Photosystem II core proteins. J. Biol. Chem. 280, 33679–33686. 10.1074/jbc.M505729200 16040609

[B233] Vassiliev S. Zaraiskaya T. Bruce D. (2012). Exploring the energetics of water permeation in photosystem II by multiple steered molecular dynamics simulations. Biochim. Biophys. Acta 1817, 1671–1678. 10.1016/j.bbabio.2012.05.016 22683291

[B234] Vener A. V. (2007). Environmentally modulated phosphorylation and dynamics of proteins in photosynthetic membranes. Biochim. Biophys. Acta 1767, 449–457. 10.1016/j.bbabio.2007.11.007 17184728

[B235] Vener A. V. Harms A. Sussman M. R. Vierstra R. D. (2001). Mass spectrometric resolution of reversible protein phosphorylation in photosynthetic membranes of *Arabidopsis thaliana*. J. Biol. Chem. 276, 6959–6966. 10.1074/jbc.M009394200 11113141

[B236] Walleczek J. Martin T. Redl B. Stofflermeilicke M. Stoffler G. (1989). Comparative cross-linking study on the 50S ribosomal subunit from *Escherichia coli*. Biochemistry 28, 4099–4105. 10.1021/bi00435a071 2665813

[B237] Wang L. W. Chance M. R. (2011). Structural mass spectrometry of proteins using hydroxyl radical based protein footprinting. Anal. Chem. 83, 7234–7241. 10.1021/ac200567u 21770468 PMC3184339

[B238] Wegener K. M. Bennewitz S. Oelmüller R. Pakrasi H. B. (2011). The Psb32 protein aids in repairing photodamaged Photosystem II in the cyanobacterium *Synechocystis* 6803. Mol. Plant 4, 1052–1061. 10.1104/pp.114.253336 21653280

[B239] Wegener K. M. Nagarajan A. Pakrasi H. B. (2015). An atypical *psbA* gene encodes a sentinel D1 protein to form a physiologically relevant inactive Photosystem II complex in cyanobacteria. J. Biol. Chem. 290, 3764–3774. 10.1074/jbc.M114.604124 25525275 PMC4319040

[B240] Wegener K. M. Welsh E. A. Thornton L. E. Keren N. Jacobs J. M. Hixson K. K.. (2008). High sensitivity proteomics assisted discovery of a novel operon involved in the assembly of Photosystem II, a membrane protein complex. J. Biol. Chem. 283, 27829–27837. 10.1074/jbc.M803918200 18693241

[B241] Weisbrod C. R. Chavez J. D. Eng J. K. Yang L. Zheng C. Bruce J. E. (2013). *In vivo* protein interaction network identified with a novel real-time cross-linked peptide identification strategy. J. Proteome Res. 12, 1569–1579. 10.1021/pr3011638 23413883 PMC3925062

[B242] Welkie D. Zhang X. Markillie M. L. Taylor R. Orr G. Jacobs J. (2014). Transcriptomic and proteomic dynamics in the metabolism of a diazotrophic cyanobacterium, *Cyanothece* sp. PCC 7822 during a diurnal light-dark cycle. BMC Genomics 15:1185 10.1186/1471-2164-15-1185 25547186 PMC4320622

[B243] Wen J. Z. Zhang H. Gross M. L. Blankenship R. E. (2009). Membrane orientation of the FMO antenna protein from *Chlorobaculum tepidum* as determined by mass spectrometry-based footprinting. Proc. Natl. Acad. Sci. U.S.A. 106, 6134–6139. 10.1073/pnas.0901691106 19339500 PMC2669346

[B244] Wetz K. Habermehl K.-O. (1979). Topographical studies on poliovirus capsid proteins by chemical modification and cross-linking with bifunctional reagents. J. Gen. Virol. 44, 525–534. 10.1099/0022-1317-44-2-525 230294

[B245] Whitelegge J. P. (2013). Integral membrane proteins and bilayer proteomics. Anal. Chem. 85, 2558–2568. 10.1021/ac303064a 23301778 PMC3664232

[B246] Whitelegge J. P. Gundersen C. B. Faull K. F. (1998). Electrospray-ionization mass spectrometry of intact intrinsic membrane proteins. Protein Sci. 7, 1423–1430. 9655347 10.1002/pro.5560070619 PMC2144037

[B247] Xu H. Freitas M. A. (2009). MassMatrix: a database search program for rapid characterization of proteins and peptides from tandem mass spectrometry data. Proteomics 9, 1548–1555. 10.1002/pmic.200700322 19235167 PMC2759086

[B248] Yang B. Wu Y.-J. Zhu M. Fan S.-B. Lin J. Zhang K.. (2012). Identification of cross-linked peptides from complex samples. Nat. Methods 9, 904–909. 10.1038/nmeth.2099 22772728

[B249] Yang M.-K. Yang Y.-H. Chen Z. Zhang J. Lin Y. Wang Y. (2014). Proteogenomic analysis and global discovery of posttranslational modifications in prokaryotes. Proc. Natl. Acad. Sci. U.S.A. 111, E5633–E5642. 10.1073/pnas.1412722111 25512518 PMC4284577

[B250] Yao D. C. I. Brune D. C. Vavilin D. Vermaas W. F. J. (2012a). Photosystem II component lifetimes in the cyanobacterium *Synechocystis* sp. strain PCC 6803: Small Cab-like proteins stabilize biosynthesis intermediates and affect early steps in chlorophyll synthesis. J. Biol. Chem. 287, 682–692. 10.1074/jbc.M111.320994 22090028 PMC3249123

[B251] Yao D. C. I. Brune D. C. Vermaas W. F. J. (2012b). Lifetimes of photosystem I and II proteins in the cyanobacterium *Synechocystis* sp. PCC 6803. FEBS Lett. 586, 169–173. 10.1016/j.febslet.2011.12.010 22197103

[B252] Zak E. Norling B. Maitra R. Huang F. Andersson B. Pakrasi H. B. (2001). The initial steps of biogenesis of cyanobacterial photosystems occur in plasma membranes. Proc. Natl. Acad. Sci. U.S.A. 98, 13443–13448. 10.1073/pnas.241503898 11687660 PMC60890

[B253] Zhang H. Liu H. Blankenship R. E. Gross M. L. (2016). Isotope-encoded carboxyl group footprinting for mass spectrometry-based protein conformational studies. J. Am. Soc. Mass Spectrom. 27, 178–181. 10.1007/s13361-015-1260-5 26384685 PMC4688080

[B254] Zhang H. Liu H. Niedzwiedzki D. M. Prado M. Jiang J. Gross M. L.. (2014). Molecular mechanism of photoactivation and structural location of the cyanobacterial orange carotenoid protein. Biochemistry 53, 13–19. 10.1021/bi401539w 24359496 PMC3963514

[B255] Zheleva D. Sharma J. Panico M. Morris H. R. Barber J. (1998). Isolation and characterization of monomeric and dimeric CP47-reaction center Photosystem II complexes. J. Biol. Chem. 273, 16122–16127. 10.1074/jbc.273.26.16122 9632665

